# Precipitation, Not Land Use, Primarily Determines the Composition of Both Plant and Phyllosphere Fungal Communities

**DOI:** 10.3389/ffunb.2022.805225

**Published:** 2022-07-07

**Authors:** Hannah I. Dea, Abigail Urban, Anna Kazarina, Gregory R. Houseman, Samantha G. Thomas, Terry Loecke, Mitchell J. Greer, Thomas G. Platt, Sonny Lee, Ari Jumpponen

**Affiliations:** ^1^ Division of Biology, Kansas State University, Manhattan, KS, United States; ^2^ Department of Biological Sciences, Wichita State University, Wichita, KS, United States; ^3^ Kansas Biological Survey and Center for Ecological Research, University of Kansas, Lawrence, KS, United States; ^4^ Environmental Studies Program, University of Kansas, Lawrence, KS, United States; ^5^ Department of Agriculture and Nutrition Science, Southern Utah University, Cedar City, UT, United States

**Keywords:** phyllosphere, fungal communities, plant communities, plant-fungal associations, precipitation gradient, environmental gradient, land use history, land use legacy

## Abstract

Plant communities and fungi inhabiting their phyllospheres change along precipitation gradients and often respond to changes in land use. Many studies have focused on the changes in foliar fungal communities on specific plant species, however, few have addressed the association between whole plant communities and their phyllosphere fungi. We sampled plant communities and associated phyllosphere fungal communities in native prairie remnants and post-agricultural sites across the steep precipitation gradient in the central plains in Kansas, USA. Plant community cover data and MiSeq ITS2 metabarcode data of the phyllosphere fungal communities indicated that both plant and fungal community composition respond strongly to mean annual precipitation (MAP), but less so to land use (native prairie remnants vs. post-agricultural sites). However, plant and fungal diversity were greater in the native remnant prairies than in post-agricultural sites. Overall, both plant and fungal diversity increased with MAP and the communities in the arid and mesic parts of the gradient were distinct. Analyses of the linkages between plant and fungal communities (Mantel and Procrustes tests) identified strong correlations between the composition of the two. However, despite the strong correlations, regression models with plant richness, diversity, or composition (ordination axis scores) and land use as explanatory variables for fungal diversity and evenness did not improve the models compared to those with precipitation and land use (ΔAIC < 2), even though the explanatory power of some plant variables was greater than that of MAP as measured by R^2^. Indicator taxon analyses suggest that grass species are the primary taxa that differ in the plant communities. Similar analyses of the phyllosphere fungi indicated that many plant pathogens are disproportionately abundant either in the arid or mesic environments. Although decoupling the drivers of fungal communities and their composition – whether abiotic or host-dependent – remains a challenge, our study highlights the distinct community responses to precipitation and the tight tracking of the plant communities by their associated fungal symbionts.

## Introduction

Aerial plant photosynthetic tissues – the phyllosphere – are among the most extensive microbial habitats on Earth (Morris et al., 2002). This habitat can be oligotrophic and exposed to rapid fluctuations in environmental conditions including shifts in temperature, humidity, and radiation (Lindow and Brandl, 2003). Yet, the phyllosphere represents a diverse ecosystem (Lindow and Leveau, 2002), colonized by hyperdiverse communities of bacteria, archaea, virus, protists, and fungi all living on (epiphytes) and within (endophytes) the leaves (Jumpponen and Jones, 2009; Martiny et al., 2011; Vorholt, 2012; Laforest-Lapointe & Whitaker, 2019). These diverse communities drive ecosystem function (Song et al., 2017; Laforest-Lapointe and Whitaker, 2019) and can contribute to nitrogen cycling by fixing nitrogen *in situ* (Furnkranz et al., 2008). Phyllosphere communities can also affect plant fitness and productivity (Davison, 1988; Schauer and Kutchera, 2011) through their modulation of stress tolerance (Vorholt, 2012) or pathogen resistance (Innerebner et al., 2011). Further, the phyllosphere communities may drive plant community dynamics (Aschehough et al., 2014; Whitaker et al., 2017; Laforest-Lapointe and Whitaker, 2019) thereby linking the phyllosphere communities to plant communities and their productivity (Laforest-Lapointe et al., 2017).

Phyllospheres are clearly important for ecosystem function and as a hotspot for microbial diversity (Arnold and Lutzoni, 2007; Laforest-Lapointe et al., 2017). Foliar fungi are among the most diverse members that can impact plant productivity and physiology within the phyllosphere (Saikkonen et al., 1998; Rodriguez et al., 2009; Meyer and Leveau, 2012; Zahn and Amend, 2019). These fungi presumably occupy photosynthetic tissues of all species and in all divisions of land plants (Bacon and White, 2000). While present in the foliage, these communities include taxa that are directly and functionally associated with the plant tissues (*e.g.*, pathogens, foliar parasites or endophytes) as well as those that may be observable on these tissues but neither penetrate the cuticle nor directly functionally interact with the host plant (*i.e.*, epiphytes that may utilize nutrients available on the foliar surfaces but never cross the cuticular barrier) (see Gomez et al., 2018). The foliar fungal communities may be more sensitive to environmental factors than those of bacteria (Bernard et al., 2021) or ectomycorrhizal fungi (Bowman and Arnold, 2021). A recent study of *Hibiscus tiliaceus* trees in Hawaii (Bernard et al., 2021) reported that while bacterial community composition was better explained by the plant organ macrohabitat, location within a steep environmental gradient better predicted variation in fungal community composition (see also Zimmerman and Vitousek, 2012). Similarly, Bowman and Arnold (2021) concluded that while the distribution of ectomycorrhizal fungi was mainly constrained by dispersal, foliar fungi were more constrained by climate factors such as mean annual precipitation and mean annual temperature. These studies exemplify the value of studying steep environmental gradients as a means to better understand how environmental variation influences the composition and assembly of fungal communities (Fraser et al., 2015; Rudgers et al., 2021).

In addition to the environment, communities can be impacted by a variety of human factors. The anthropogenic conversion of natural ecosystems presents a substantial threat to biodiversity (Foley et al., 2005; Newbold et al., 2015; Perreault and Laforest-Lapointe, 2021). Human land-use, including agriculture and silviculture, can have long-lasting legacies wherein the altered ecosystem attributes persist after cessation of human land-use (Dupouey et al., 2002; Foster et al., 2003; Flinn et al., 2005; McLauchlan, 2006; Cramer et al., 2008). These systems struggle with the establishment of native plant communities after the human land-use abandonment (Kuussaari et al., 2009; Moreno-Mateos et al., 2017). For example, compared to systems that have no history of human use, former agricultural lands may possess altered soils, non-native plant communities, and other distinct ecosystem properties for decades and even millennia following farm abandonment (Bellemere et al., 2002; Dupouey et al., 2002; Flinn and Marks, 2007). Similarly, agricultural land-use history can reduce soil-inhabiting fungal diversity and result in communities distinct from those in native remnants that have never been used for production agriculture (Oehl et al., 2003; Wagg et al., 2018; Turley et al., 2020). Phyllosphere communities may be less responsive to edaphic factors as they do not directly interact with the soil matrix, whose biogeochemical attributes may strongly influence soil-inhabiting communities. Consistent with this, community composition of the foliar fungi often reflects climatic factors (Bowman and Arnold, 2021) such as mean annual temperature and precipitation (Oita et al., 2021), rather than variation in soil properties. This is particularly true if phyllosphere communities are assessed broadly and include casual epiphytes that may only utilize readily available resources on the leaf surfaces. Even within the phyllosphere, controls of communities in foliar compartments may differ, as the communities of leaf epiphytes and endophytes may be shaped by distinct environmental controls (Gomes et al., 2018).

Although plant and fungal communities and their responses to environmental gradients have been targets of many studies, analyses to better establish linkages among them are still rare. Large-scale studies have reported correlations between plant and fungal richness (Arnold and Lutzoni, 2007; Tedersoo et al., 2020) that may often stem from collinearities and/or correlations between plants and associated fungal communities. In this contribution, we attempt to concurrently dissect plant communities as well as those fungal communities that occupy their photosynthetic tissues. Many studies thus far have focused on diversity at the local scales (Allan et al., 2014; Newbold et al., 2015) but neglected changes at larger spatial scales. We utilized the steep precipitation gradient in the state of Kansas (USA) located in the Great Plains to assess how plant communities and their foliar fungal communities may respond to this precipitation gradient, how the plant and fungal communities may differ across two distinct historic land uses (post-agricultural sites and native remnant prairies), whether the communities within these two historic land uses respond differently to precipitation, and how the plant communities and their foliar communities may be linked to each other. Agricultural systems that have a history of intensive human land-use are a common focus of restoration efforts but how post-agricultural fields compare to native prairie remnants remains unclear particularly for fungal communities that occupy photosynthetic tissues. We hypothesized that 1) plant and their phyllosphere fungal communities increase in richness, evenness, and diversity with increasing the mean annual precipitation; 2) post-agricultural sites – as a result of their previous intensive agricultural use – have a lower richness and diversity as well as distinct communities when compared to native prairie sites; 3) native prairie remnants and post-agricultural sites differ in their responses to the precipitation gradient such that richness, diversity, and evenness in the remnant prairie sites respond more strongly to precipitation than post-agricultural prairies; and 4) fungal communities correlate with plant communities in diversity and composition. We emphasize that the approaches linking aboveground plant diversity with fungal richness and diversity are rare (Cho et al., 2017) and that studies across land-use systems and plant diversity are required to enable sound recommendations for sustainable land-use (Monkai et al., 2017).

## Materials and Methods

### Study Sites and Sampling

**Figure 1:**
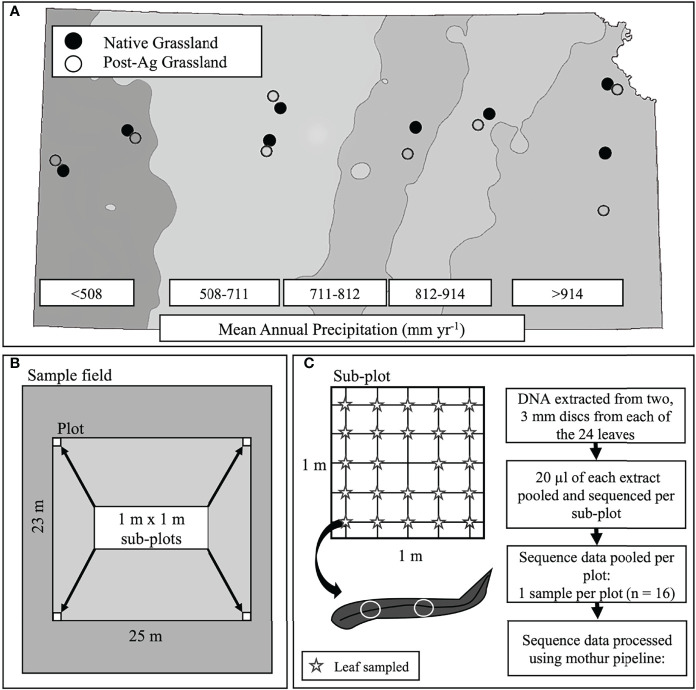
A schematic of the experimental design including site locations across the precipitation gradient of Kansas, USA with mean annual precipitation (MAP) bands indicated with shading. Points indicate sampled sites (open circles – post-agricultural sites; solid circles – native prairie remnants) **(A)**, plot and sub-plot layout within each site **(B)**, and leaf sampling within each sub-plot and subsequent sample and sequence data processing **(C)**.

During the summer of 2019, we located eight post-agricultural and eight native remnant prairie sites along the steep precipitation gradient in Kansas for a total of sixteen sites with mean annual precipitation (MAP) ranging from 455.74 mm yr^-1^ to 1040.46 mm yr^-1^ and mean annual temperature (MAT) ranging from 11.31°C to 13.30°C (**Figure 1A**; **Table 1**). We specifically targeted sites that would represent the precipitation gradient while stratifying our sampling to similar soil types along the Kansas River watershed, which runs across the state east-to-west at approximately the 39^th^ parallel (longitude ranging from 095° 16’ 21.42”W to 101° 47’ 06.31”W). The chosen sites had similar edaphic characteristics and occurred in a similar landscape position (*e.g.*, toe slope or flood slope terrace). Additionally, we sampled sites alternating between the arid and mesic ends of the gradient to minimize the potential for temporally confounding factors in a sampling that required a little over three months (June 12^th^–September 18^th^, 2019).

**Table 1:** Site details including site identifiers, land use history (native prairie remnant or post-agricultural site), coordinates, mean annual temperature (MAT), mean annual precipitation (MAP) (acquired from PRISM Climate Group at Oregon State University; https://prism.oregonstate.edu/), soil type (as defined in USDA Natural Resources Conservation Service SSURGO database) and Sequence Read Archive accession under BioProject PRJNA795108.

Sample	Land Use	Coordinates (DMS)	MAT (°C)	MAP (mm yr^-1^)	Soil Type	Accession
TRB_N	Native	38° 28’ 10.17”N 101° 46’ 56.05”W	11.31	455.74	Richfield (1761)	SAMN24688330
TRB_P	Post-ag	38° 28’ 18.95”N 101° 47’ 06.31”W	11.31	455.74	Richfield (1761)	SAMN24688331
SVR_N	Native	38° 52’ 27.35”N 100° 59’ 03.49”W	11.68	477.00	Ulysses (1857)	SAMN24688326
SVR_P	Post-ag	38° 51’ 58.07”N 100° 59’ 43.36”W	11.68	477.00	Ulysses (1857)	SAMN24688327
HAY_N	Native	38° 50’ 07.50”N 099° 18’ 12.15”W	12.28	604.68	Harney (2612)	SAMN24688313
HAY_P	Post-ag	38° 50’ 40.11”N 099° 18’ 58.83”W	12.23	602.91	Armo (2518)	SAMN24688314
RKS_N	Native	39° 10’ 29.79”N 099° 09’ 00.90”W	11.90	634.91	Heizer-Harney-Brownell-Bogue-Armo (s2536)	SAMN24688323
RKS_P	Post-ag	39° 09’ 50.84”N 099° 09’ 43.72”W	11.90	634.91	Heizer-Harney-Brownell-Bogue-Armo (s2536)	SAMN24688324
TLI_N	Native	38° 58’ 11.13”N 097° 28’ 08.50”W	13.30	760.86	Hord (3755)	SAMN24688318
TLI_P	Post-ag	38° 45’ 58.94”N 097° 34’ 26.57”W	13.21	781.65	McCook (2347)	SAMN24688319
KNZ_N	Native	39° 06’ 20.06”N 096° 36’ 36.65”W	12.74	850.63	Reading (7174)	SAMN24688315
KNZ_P	Post-ag	39° 06’ 12.33”N 096° 36’ 15.92”W	12.53	860.23	Reading (7170)	SAMN24688316
LVN_N	Native	39° 15’ 31.46”N 094° 58’ 42.46”W	12.55	1003.32	Shelby-Sharpsburg (s2389)	SAMN24688320
LVN_P	Post-ag	39° 15’ 38.52”N 095° 00’ 57.49”W	12.58	997.13	Pawnee-Grundy (s2386)	SAMN24688321
EKS_N	Native	38° 10’ 52.44”N 095° 16’ 21.42”W	13.23	1040.46	Kenoma-Olpe (8780)	SAMN24688311
EKS_P	Post-ag	38° 10’ 52.32”N 095° 16’ 27.12”W	13.23	1040.46	Kenoma (8875)	SAMN24688312

At each site, we established a 25 m x 23 m plot. Within each plot, we established a 1m x 1m subplot in each of the four corners for a total of four subplots (**Figure 1B**). The GPS location was recorded using an Eos Arrow 100 Submeter GNSS Receiver (Eos Positioning Systems^®^, Inc., Terrebonne Canada), and the subplot corners marked to permit sampling of the plant and fungal communities even if the teams sampling plants and fungi could not sample simultaneously. Within each subplot, we identified all plant individuals to species (**Supplementary Tables 1** and **2**). When this was not possible, we reverted to genus (*e.g*., species within *Cyperus* and *Carex* were pooled). We quantified plant abundance by comparing the canopy cover of each species or morphospecies within the subplot to a cover card that was marked with cover percentages of various sizes. For species that occupied large areas, we counted the number of cover card areas required to match the cover of a particular species. These values were then summed to get the subplot cover for that species. We recorded values to the nearest percentage or fraction of a percentage for values below 1%. We then measured the modal height of each species in 10-cm height classes in the subplot. Plant height varied strongly across the sites because of the differences in the plant communities (short to tallgrass prairie) and the timing of sampling (plant phenology). Because we used a visual comparison of the cover card to plant cover, there was a potential for error if the cover card was held at different distances within and among observers due to the foreshortening effect (distance between eye, cover card, and plant height could appear to have different values). To minimize this problem, all observers held the cover card in the same way each time. We then adjusted for differences in the eye-hand relationships of each observer and differences in plant height. To do this, each observer measured swatches of a known size at various heights in the lab. These data were used to calculate an observer-specific plant height correction that was applied to final cover values (Watson et al., 2021).

In order for fungal community samples to reflect the co-occurring plant communities, we sampled fungal communities using a systematic gridline intersection sampling. In each of the four subplots, we placed a 1 m x 1 m quadrate gridded 20 cm apart for a total of 25 intersects (**Figure 1C**). At each intersect, we lowered a wooden dowel rod and excised the first, topmost leaf the dowel rod touched and placed the leaves individually in sterile plastic bags skipping the middle intersect for a total of 24 leaf samples within each subplot and 96 for each full plot (single land use within a precipitation band). Samples were placed on ice in a cooler and processed in the laboratory within 24 hours of collection. Our sampling allowed us to capture representative plant taxa and minimize plant height bias due to the spatial (four representative 1 m x 1 m subplots) and temporal (samples taken throughout the growing season) heterogeneity of our sampling efforts.

### DNA Extraction, PCR Amplification, and Sequencing of Fungal Communities

To extract total environmental DNA from the sampled leaves, we excised two 3 mm disks from each of the 24 leaves from each subplot using sterile Ted Pella Biopsy Punches (Ted Pella Inc., Redding, CA). We followed the ThermoFisher Phire Plant Direct kit manufacturer’s protocol (ThermoScientific, Pittsburg USA) to isolate total DNA from the leaf tissues. In brief, we suspended the two leaf disks in 40 µl of dilution buffer and crushed the leaf disks with round tip forceps. We then pooled 20 µl of each of the twenty-four extractions for each subplot into one representative sample (4 subsamples for each of the 16 plots).

To choose the optimal dilution for PCR-amplification, we diluted the extracts (10^0^ - 10^-3^) in sterile molecular grade RNA- and DNA-free water. In pilot reactions, the 10^-2^ dilution consistently and reliably produced PCR-amplicons and was chosen for library preparation. To analyze the fungal communities, we PCR-amplified the Internal Transcribed Spacer (ITS2) of the ribosomal RNA gene (Schoch et al., 2012) with the forward fITS7 (Ihrmark et al., 2012) and reverse ITS4 (White et al., 1989) primers in 50 µl duplicate PCR reactions. Both the forward and reverse primers included a sample specific 12 bp Molecular Identifier DNA (MID) (Caporaso et al., 2012). The volumes and final concentrations of reagents were as follows: 2.5 µl forward and reverse primer (0.5 µM), 5 µL 10^-2^ diluted template DNA, 25 µL of 2X Phire Plant Direct PCR Master Mix, and 17.5 µL molecular grade water. The PCR reactions included an initial denaturing step for 30 s (98°C) and were followed by 30-35 cycles of 10 s of denaturing (98°C); 30 s of annealing (54°C); 1 min of extension (72°C); and concluding with a 9 min final extension (72°C). When 30 cycles did not amplify, we repeated the reactions with 35 cycles. The PCR reactions included sterile molecular grade RNA- and DNA-free water as a negative control and a fungal mock community as a positive control to calculate internal sequencing error as described in Mothur Standard Operation Protocol (SOP) (Kozich et al., 2013). We constructed the fungal mock community from nine fungal pure cultures that broadly represent fungal taxa (Ascomycota: *Aspergillus niger*, *Chaetomium globosum*, *Penicilium notatum* (synonym *Penicillium chrysogenum*), *Saccharomyces cerevisiae*, *Sordaria fimicola*; Basidiomycota: *Coprinopsis cinerea;* Chytridiomycota: *Phlyctochytrium acuminatum* (synonym *Spizellomyces acuminatus*); Mucoromycota: *Phycomyces blakesleeanus*, *Rhizopus stolonifer*). We extracted DNA from two-week old cultures with the DNeasy PowerSoil DNA Isolation Kit (Qiagen, Germantown, Maryland) as per the manufacturer’s protocol and equal volumes of 2 ng/µL of each extraction were pooled. We combined a total of 45 µL of each duplicate PCR-amplicon for each sample including positive and negative controls. We purified the pooled 90 µl volumes using the Mag-Bind RXNPure Clean-up system (Omega Bio-Tek Inc., NorCross, Georgia) following a modified manufacturer’s protocol with a 1:1 ratio of PCR product to AMPure solution and two rinse steps with 80% ethanol. A total of 250 ng of purified DNA per sample was pooled into one. As the negative control did not yield quantifiable amplicons, the whole 90 µl volume was included in the pool.

Illumina adapters and indices were added using four PCR cycles, KAPA Hyper Prep Kit (Roche, Pleasenton, CA USA), and 0.5 µg starting DNA. The library was sequenced (2 x 300 cycles) using the Illumina MiSeq Personal Sequencing System at the Integrated Genomics Facility (Kansas State University, Manhattan KS USA). The sequence data are available through the Sequence Read Archive under BioProject PRJNA795108; BioSamples SAMN24688311- SAMN24688331.

### Sequence Data Processing

The sequence data were processed using the mothur pipeline (v. 1.44.3; Schloss et al., 2009) following mainly the MiSeq standard operating protocol to generate ASV (Amplified Sequence Variant) and OTU (Operational Taxonomic Unit) data. In brief, the sequence data for each experimental unit were identified by Molecular Identifier DNAs (MIDs; Caporaso et al., 2012), extracted from the paired-end.fastq files and assembled into contigs. Sequences with more than 1 bp difference with the primers, without an exact match to the MIDs, or with long homopolymers (maxhomop = 8) were omitted. Since the four sub-plot samples were not independent but rather represented one site, we pooled the libraries to one per plot (for a total of 16 experimental units). We considered this necessary to avoid pseudo-replication, as the adjacent subplots would not represent true replicates of the main effects (mean annual precipitation and land-use) in our models. Sequences were truncated to the length equal to the shortest high-quality read (237 bp excluding primers and MIDs), pre-clustered (Huse et al., 2010), and potential chimeras identified (UCHIME; Edgar et al., 2011) and culled. The remaining sequences were assigned to taxon affinities using the Naïve Bayesian Classifier (Wang et al., 2007) and the UNITE taxonomy reference (Abarenkov et al., 2021). Non-target reads (those with no match in the UNITE-curated INSD or assigned to Protista and Plantae) were removed from further analyses. The quality-screened sequences were assigned to ASVs and subsequently clustered to OTUs at 97% similarity using vsearch (Rognes et al., 2016). Rare ASVs and OTUs represented by fewer than ten reads were removed (Brown et al., 2015; Oliver et al., 2015).

### Data Analyses

Fungal communities were analyzed as both ASVs and OTUs. Consistent with other analyses comparing ASVs and OTUs (Glassman and Martiny, 2018; Tipton et al., 2021; Tawidian et al., 2021), our analyses also yielded comparable results. As a result, we present the OTU analyses here, whereas the ASV analyses are available as a supplement (**Supplementary File 1**). We iteratively (100 iterations) calculated observed (S_Obs_) richness, Shannon’s diversity (H’), and evenness based on Shannon’s diversity (E_H_) using the mothur pipeline (v. 1.44.3; Schloss et al., 2009). We subsampled the sequence data to 90000 sequences per sample, as recommended in (Gihring et al., 2012) to avoid biased comparisons of estimators in samples with unequal sequence yields.

Statistical analyses were performed using program R (R Core Team 2021-2022). We used multiple linear regression analyses to predict plant and fungal richness, diversity, evenness, sample scores of the first and second PCoA axes (*i.e.*, sample coordinates in ordination space), and adjusted Floristic Quality Index (FQI_adj_) responses to Mean Annual Precipitation (MAP) normalized around the mean (730.01 mm yr^-1^), land use history (LU), and their interaction using the “lm()” function in program R. For plant communities, we relativized all cover values by total plant cover. Plant community diversity metrics were calculated using the “community structure()” function in the “codyn” package in program R (v. 2.0.5; Hallett et al., 2020). We used E_var_ as our metric for plant communities because it is least sensitive to differences in species richness (Smith and Wilson, 1996). We also estimated FQI_adj_ in each plot (Freyman et al., 2016). FQI_adj_ was initially developed by Wilhelm and Kane County (1977) and is a commonly used conservation indicator that provides a numerical value for the ecological value for restoration success of a site and uses ratios between native and total species richness (see Watson et al., 2021 for further details). We visually evaluated residuals to confirm that they did not present any blatant violations of assumptions of linear regression analyses and performed outlier analyses. Three samples (LVN_N ASV and OTU S_obs_; TRB_N plant H’ and plant PCoA axis 2; and TRB_P plant FQI_adj_) represented potential outliers (values greater than 2 standard deviations from the mean). We analyzed our data both with and without these data points to determine if they drove patterns in our results.

To determine if any other explanatory variables were superior to MAP in explaining variation in plant and fungal community estimators, we replaced MAP with geographic distance (change in longitude) for responses of plant (richness, diversity, evenness, FQI _adj_, or first PCoA axis) and fungal (richness, diversity, evenness, or first PCoA axis) community estimators in models combining it with LU and their interaction as predictors. We also compared models with MAP replaced by plant community estimators (richness, diversity, evenness, FQI _adj_, or first PCoA axis) as explanatory variables in models combining LU and interaction terms for responses in fungal community estimators (richness, diversity, evenness, or first PCoA axis). We compared the change in AIC values to identify the superior models (ΔAIC > 2) as described by Burnham and Anderson (2004).

To visualize and infer compositional differences within plant and fungal communities, we calculated pairwise Bray-Curtis distances and visualized these data with Principal Coordinates Analysis (PCoA) using function “ordinate()” (method = ‘PCoA’) in R package “phyloseq” (v. 1.38.0; McMurdie and Holmes, 2013). To control for library size, i.e. sequencing depth, we rarefied our community abundance data to 95000 sequences for ASVs and 99000 sequences for OTUs using function “rarefy_even_depth()” in “phyloseq” (v. 1.38.0; McMurdie and Holmes, 2013). To test for the main and interactive effects of land use history and MAP (grouped into “arid” for 455.7 – 634.9 mm yr^-1^ and “mesic” for 760.9 – 1040.5 mm yr^-1^), we used a non-parametric permutational analysis of variance (PERMANOVA) on the Bray-Curtis distance matrix using function “adonis()” in “vegan” (v. 2.5-7; Oksanen et al., 2020). We further analyzed community composition using a constrained ordination, distance-based redundancy analyses, which allowed for use of MAP as a continuous explanatory variable, using main effects of MAP, MAT, longitude, and LU for plant communities with the addition of plant first PCoA axis to explain variation in fungal communities using function “ordinate()” (method = ‘CAP’) in R package “phyloseq” (v. 1.38.0; McMurdie and Holmes, 2013) and inferred differences using function “anova()”. We tested the null hypothesis that experimental units have similar multivariate dispersion using the “betadisper()” function in “vegan” (v. 2.5-7; Oksanen et al., 2020). To determine if any plants, ASVs, or OTUs were disproportionately abundant in the arid or mesic precipitation habitats, we used indicator species analyses with the “multipatt()” function in R package “indicspecies” (v. 1.7.12; De Caceres and Legendre, 2009) on the 50 most abundant plants, 100 most abundant OTUs, and 200 most abundant ASVs and corrected P-values for multiple testing using function “p.adjust()” with false discovery rate (FDR) method in program R. To test the association between plant and fungal communities, we used Mantel tests to compare Bray-Curtis distance matrices using function “mantel()” in “vegan” (v. 2.5-7; Oksanen et al., 2020). Similarly, to test the association of geographic distance with plant and fungal communities, we calculated the pairwise Haversine distance between sample coordinates using “distm()” function in R package “geosphere” (v. 1.5-14; Hijmans, 2021) and used Mantel tests to compare with Bray-Curtis distances. Additionally, to compare the plant and fungal PCoA ordinations, we used Procrustes analyses of the plant and fungal PCoA ordinations using function “procrustes()” in “vegan” (v. 2.5-7; Oksanen et al., 2020).

## Results

### Community Descriptions

In the 16 total samples from eight native remnant prairies and eight post-agricultural sites, we observed a total of 160 plant species representing a total of 36 families. The Family Poaceae was dominant (34 species and 62.7% of total cover), followed by Family Asteraceae (38 species and 19.0% total cover), Family Fabaceae (21 species and 6.4% total cover), Family Amaranthaceae (2 species and 3.3% total cover), Cyperaceae (3 species and 1.7% total cover), and Family Anacardiaceae (1 species and 1.5% total). Other families represented < 1% of the total cover (**Supplementary Figure 1A**). The plant community cover data and taxonomic information are listed in **Supplementary Tables 1** and **2**.

Following quality control and removal of rare sequences, we retained a total of 3,328,786 high quality sequences that clustered into 4,385 OTUs. The sequencing yields ranged from 99,025 to 366,866 per sample with a mean yield of 208,049 ± 92,819.02 (SD). The OTUs, their observed frequencies, and taxonomic assignments are listed in **Supplementary Tables 3** and **4**.

Our data were dominated by the Phylum Ascomycota (63.6% sequences and 50.4% OTUs), the Phylum Basidiomycota (16.0% sequences and 18.1% OTUs), and a fairly large portion of unidentified taxa (18.2% sequences and 17.6% OTUs), followed by the Phylum Glomeromycota (1.3% sequences and 7.4% OTUs), Chytridiomycota (0.4% sequences and 4.3% OTUs), and several Phyla that made up <1% of sequences and OTUs (Mortierellomycota, Mucoromycota, Kickxellomycota, Rozellomycota, Olpidiomycota, Entorrhizomycota, Aphelidiomycota, Entomophthoromycota, Aphelidiomycota, Calcarisporiellomycota, and Blastocladiomycota) (following Tedersoo et al., 2018). Relative abundance of fungal orders can be found in **Supplementary Figure 1C**. OTUs were assigned to a total of 774 genera. A large proportion of the OTUs (2,078 OTUs) were not assigned to a genus (47.3%). Among those with genus level assignments, the most abundant were *Alternaria* with 7 OTUs (4% sequences and > 0.2% of all OTUs), followed by *Cladosporium* with 2 OTUs (3.5%) and *Dissoconium* with 8 OTUs (2.8% sequences). The ten most abundant genera were common phyllosphere inhabitants including *Alternaria*, *Dissoconium*, *Phaeosphaera*, *Puccinia*, *Fusarium*, *Blumeria*, and *Aureobasidium*.

### Alpha Diversity and Regression Analyses

**Figure 2:**
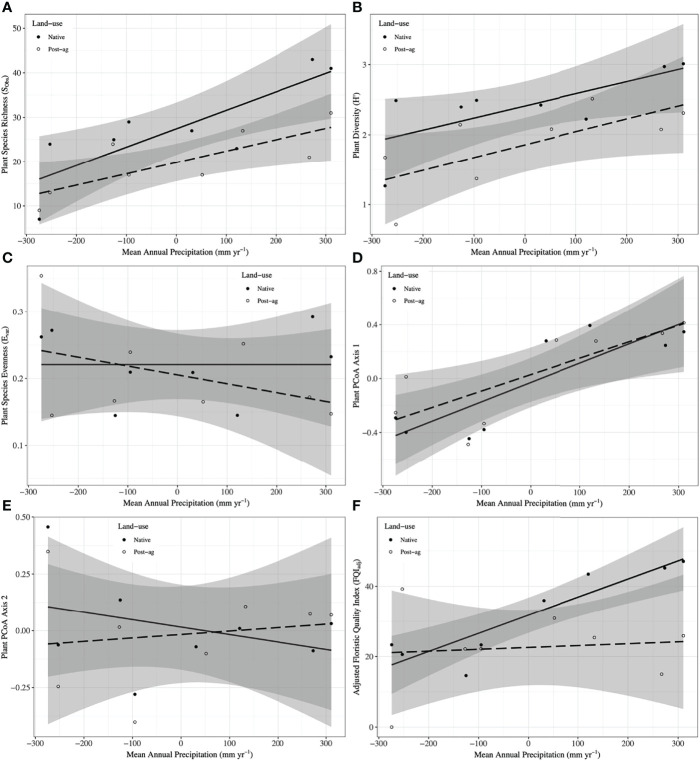
Plant community responses to mean annual precipitation (MAP) normalized around the mean precipitation (730.01 mm yr^-1^) in native prairie remnants (solid line and filled symbols) and post-agricultural sites (dashed line and open symbols). Models predict observed species richness (S_Obs_) **(A)**, Shannon diversity (H’) **(B)**, evenness (E_var_) **(C)**, Adjusted Floristic Quality Index (FQI_adj_) **(D)**, PCoA Axis 1 scores **(E)**, PCoA Axis 2 scores **(F)**. The shaded areas represent 95% confidence intervals around the model predictions.

**Figure 3:**
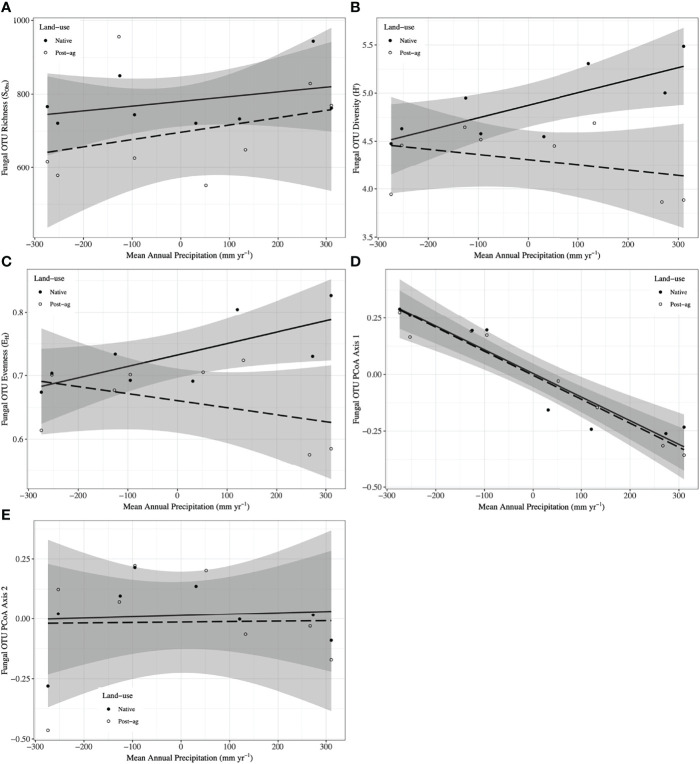
Fungal Operational Taxonomic Unit (OTU) responses to mean annual precipitation (MAP) normalized around the mean precipitation (730.01 mm yr^-1^) in native prairie remnants (solid line and filled symbols) and post-agricultural sites (dashed line and open symbols). Models predict observed species richness (S_Obs_) **(A)**, Shannon diversity (H’) **(B)**, evenness (E_H_) **(C)**, PCoA Axis 1 scores **(D)**, PCoA Axis 2 scores **(E)**. The shaded areas represent 95% confidence intervals around the model predictions.

Our regression model — using MAP normalized around the mean precipitation (730.01 mm yr^-1^), LU, and their interaction as predictors — predicted plant richness and explained a large proportion of its variation (**Table 2**). Plant richness increased with MAP, and prairie remnants had greater plant richness than the post-agricultural sites. We observed no evidence for an interaction between MAP and land-use suggesting that the plant richness increased similarly in both land-uses (**Table 2**; **Figure 2A**). AIC comparisons suggest that replacing MAP with geographic distance did not result in a superior model for predicting plant richness (**Supplementary Table 5**). In contrast to plant richness, our regression models poorly predicted fungal richness (S_Obs_) and explained only a small proportion of the variation. These analyses provided no evidence for fungal richness responses to MAP, LU, or their interaction (**Table 3**; **Figure 3A**). This result did not change whether or not the potential outlier (LVN_N) was excluded from the analysis (**Supplementary Table 6**). AIC comparisons suggest that plant predictors or geographic distance were not superior to MAP (**Supplementary Table 7**) except in the case of plant richness which was a better predictor for OTU richness (F_3,12_ = 2.435, R^2^_adj_ = 0.223, P = 0.115). However, in general, none of these alternative models performed well in predicting fungal richness overall.

**Table 2:** Multiple linear regression model statistics for plant community diversity, richness, evenness, and compositional estimates predicted by land use history (LU) and mean annual precipitation (MAP) normalized around the mean precipitation (730.01 mm yr^-1^) main effects and their interaction (LU x MAP) with native prairie remnants as reference (0) compared to post-agricultural sites (1).

Response	Model	Predictor	Estimate ± SE	|t-value|
Plant FQI_adj_^1^	F_3,12 =_ 4.53^*^, R^2^_adj_=0.414, AIC=123.44	**Intercept**	**31.78 ± 3.42**	**9.29** ^***^
		Land Use (LU)	–9.19 ± 4.84	–1.90^(*)^
		**MAP**	**5.16x10^-2^ ± 1.64x10^-2^**	3.16^**^
		LU x MAP	–4.63x10^-2^ ± 2.31x10^-2^	–2.00^(*)^
				
Plant Richness (S_Obs_)	F_3,12 =_ 9.99^*^, R^2^_adj_=0.643, AIC=107.85	**Intercept**	**27.44 ± 2.10**	**13.05** ^***^
		**LU**	**–7.60 ± 2.97**	**–2.56** ^*^
		**MAP**	**4.15x10^-2^ ± 1.00x10^-2^**	4.13^**^
		LU x MAP	–1.59x10^-2^ ± 1.42x10^-2^	–1.12^ns^
				
Plant Diversity (H’)^2^	F_3,12 =_ 6.24^*^, R^2^_adj_=0.512, AIC=23.61	**Intercept**	**2.41 ± 1.51x10** ^-1^	**15.96** ^***^
		**LU**	**–5.55x10^-1^ ± 2.14x10^-1^**	–2.60^*^
		**MAP**	**1.72x10^-3^ ± 7.22x10^-4^**	2.39^*^
		LU x MAP	1.02x10^-4^ ± 1.02x10^-3^	0.10^ns^
				
Plant Evenness (E_var_)	F_3,12 =_ 0.55^ns^, R^2^_adj_=–0.098, AIC= –36.28	Intercept	2.21x10^-1^ ± 2.33x10^-2^	9.50^***^
		LU	–1.55x10^-2^ ± 3.29x10^-2^	–0.47^ns^
		MAP	4.85x10^-8^ ± 1.11x10^-4^	0.00^ns^
		LU x MAP	–1.32x10^-4^ ± 1.57x10^-4^	–0.84^ns^
				
Plant PCoA Axis 1	F_3,12 =_ 8.47^**^, R^2^_adj_=0.599, AIC=2.75	Intercept	**–2.90**x10^-2^ **± 7.88x10^-2^**	**–3.78** ^ns^
		LU	5.83x10^-2^ ± 1.11x10^-1^	0.52^ns^
		**MAP**	**1.44x10^-3^ ± 3.76x10^-4^**	3.82^**^
		LU x MAP	–2.17x10^-4^ ± 5.32x10^-4^	–0.41^ns^
				
Plant PCoA Axis 2^3^	F_3,12 =_ 0.31^ns^, R^2^_adj_=–0.160, AIC=4.10	Intercept	1.60x10^-2^ ± 8.22x10^-2^	0.19^ns^
		LU	–3.27x10^-1^ ± 1.16x10^-1^	–0.28^ns^
		MAP	–3.29x10^-4^ ± 3.92x10^-4^	–0.84^ns^
		LU x MAP	4.80x10^-4^ ± 5.54x10^-4^	0.87^ns^

Ta: Statistically significant models and predictors (P<0.05) are bold-faced. Parameter estimate significances are denoted as ‘ns’ for not significant, ‘(*)’ for 0.05≤P < 0.10, ‘*’ for 0.01≤P < 0.05, ‘**’ for 0.001≤P < 0.01, and ‘***’ for P < 0.001. Response variables with outliers were analyzed with and without the identified outliers. Models shown here include potential outliers, **Supplementary Table 6** provides model details with outliers removed.^1^Contained a low potential outlier in TRB_P retained in this analysis.^2^ Contained a low potential outlier in TRB_N retained in this analysis.^3^ Contained a low potential outlier in TRB_N retained in this analysis.

**Table 3:** Multiple linear regression model statistics for fungal Operational Taxonomic Unit (OTU) community diversity, richness, evenness, and compositional estimates predicted by land use history (LU) and mean annual precipitation (MAP) normalized around the mean precipitation (730.01 mm yr^-1^) main effects and their interaction (LU x MAP) with native prairie remnants as reference (0) compared to post-agricultural sites (1).

Response	Model	Predictor	Estimate ± SE	|t-value|
OTU Richness (S_Obs_)^4^	F_3,12 =_ 1.17^ns^, R^2^_adj_=0.033, AIC=202.93	**Intercept**	**780.37 ± 41.03**	19.02^***^
		LU	–83.71 ± 58.02	–1.44^ns^
		MAP	1.29x10^-1^ ± 1.96x10^-1^	0.66^ns^
		LU x MAP	6.90x10^-2^ ± 2.77x10^-1^	0.25^ns^
				
OTU Diversity (H’)	F_3,12 =_ 6.89^*^, R^2^_adj_=0.541, AIC=13.21	**Intercept**	**4.87 ± 1.09x10^-1^**	44.63^***^
		**LU**	**5.65x10^-1^ ± 1.54x10^-1^**	–3.66^**^
		**MAP**	**1.30x10^-3^ ± 5.22x10^-4^**	2.49^*^
		**LU x MAP**	**–1.84x10^-3^ ± 7.37x10^-4^**	–2.50^*^
				
OTU Evenness (E_H_)	F_3,12 =_ 4.70^*^, R^2^_adj_=0.426, AIC= –44.69	**Intercept**	**7.32x10^-1^ ± 1.79x10^-2^**	40.95^***^
		**LU**	**–7.17x10^-2^ ± 2.53x10^-2^**	–2.84^*^
		MAP	1.80x10^-4^ ± 8.54x10^-5^	2.10^(*)^
		**LU x MAP**	**–2.90x10^-4^ ± 1.21x10^-4^**	–2.40^*^
				
OTU PCoA Axis 1	F_3,12 =_ 42.88^***^, R^2^_adj_=0.893, AIC= –30.80	Intercept	4.18x10^-3^ ± 2.76x10^-2^	0.15^ns^
		LU	**–8.34**x10^-3^ ± 3.90x10^-2^	**–**0.21^ns^
		**MAP**	**–1.05x10^-3^ ± 1.32x10^-4^**	–7.95^***^
		LU x MAP	–1.48x10^-5^ ± 1.86x10^-4^	–0.08^ns^
				
OTU PCoA Axis 2	F_3,12 =_ 0.03^ns^, R^2^_adj_= **–**0.240, AIC=0.49	Intercept	1.41x10^-2^ ± 7.34x10^-2^	0.19^ns^
		LU	–2.81x10^-2^ ± 1.04x10^-1^	–0.27^ns^
		MAP	5.64x10^-5^ ± 3.51x10^-4^	0.16^ns^
		LU x MAP	–3.72x10^-5^ ± 4.95x10^-4^	–0.08^ns^
				

Statistically significant models and predictors (P<0.05) are bold-faced. Parameter estimate significances are denoted as ‘ns’ for not significant, ‘(*)’ for 0.05≤P<0.10, ‘*’ for 0.01≤P<0.05, ‘**’ for 0.001≤P<0.01, and ‘***’ for P<0.001. Response variables with outliers were analyzed with and without the identified outliers Models shown here include potential outliers, **Supplemental Table 7** provides model details with outliers removed.4Contained a high potential outlier in LVN_N retained in this analysis.

Our regression models — using MAP normalized around the mean precipitation (730.01 mm yr^-1^), LU, and their interaction as predictors — predicted both plant and fungal diversity (H’) and explained a large proportion of the variation in both communities (**Tables 2**, **3**; **Figures 2B**, **3B**). Plant diversity increased with MAP and native prairie remnants harbored greater plant diversity than post-agricultural sites. However, we found no evidence of interaction between MAP and land-use (**Table 2**; **Figure 2B**). When the potential low outlier (TRB_N) was removed, our model explained more of the variation in plant diversity, the land-use term had a greater explanatory power, whereas MAP decreased in explanatory power (**Supplementary Table 6**). However, because the potential outlier represents the dry terminal end of the precipitation gradient, it likely represents an accurate value for the site. AIC comparisons suggested that replacing MAP with geographic distance did not result in a superior model in predicting plant diversity (**Supplementary Table 5**). There was evidence for interaction between MAP and LU in models predicting fungal diversity: fungal diversity increased with MAP in the native prairie remnants but did not significantly change with increasing MAP in post-agricultural sites. There was also evidence for a land-use main effect that indicated greater fungal diversity in native remnant prairies than post-agricultural sites (**Table 3**; **Figure 3B**). AIC comparisons suggest that FQI _adj_ and geographic distance were comparable to MAP in explaining fungal diversity (**Supplementary Table 7**)

Our regression models – using MAP normalized around the mean precipitation (730.01 mm yr^-1^), LU, and their interaction as predictors – neither predicted plant community evenness (E_var_) nor explained much of its variation (**Table 2**; **Figure 2C**). Plant community evenness was not influenced by MAP, LU, or their interaction. AIC comparisons suggest that replacing MAP with geographic distance did not result in superior model for predicting plant evenness (**Supplementary Table 5**). In contrast, our regression model predicted fungal community evenness (E_H_) and explained a considerable proportion of its variation (**Table 3**; **Figure 3C**). There was some evidence for interaction between MAP and LU. Fungal evenness seemed to increase with MAP in native prairies, but did not change in post-agricultural sites. There was also evidence for a land-use main effect indicating greater fungal evenness in native remnant prairies than post-agricultural sites (**Table 3**; **Figure 3C**). AIC comparisons suggest that FQI _adj_ and geographic distance were comparable to MAP in explaining fungal evenness (**Supplementary Table 7**)

In addition to plant richness and diversity, we estimated the adjusted Floristic Quality Index (FQI_adj_) that aims to provide a numerical measure reflecting the quality of plant communities. Our model — using MAP normalized around the mean precipitation (730.01 mm yr^-1^), LU, and their interaction as predictors — predicted plant FQI_adj_ and explained some of the variation in the model (**Table 2**; **Figure 2F**). In general, plant FQI_adj_ increased with MAP. There was also some marginal evidence suggesting that native prairie remnants had greater FQI_adj_ than post-agricultural sites. Similarly, there was some evidence for an interaction between MAP and land-use suggesting that, while the FQI_adj_ increased in native prairies, it did not in the post-agricultural sites. When the potential outlier (TRB_P) was removed, our model predicted FQI_adj_ and explained more of the variation. The marginal interactive effects became highly significant suggesting that the native prairie FQI _adj_ increased with MAP, whereas it decreased in the post-agricultural sites. However, since the two regression lines now intersect the intercept, the main effect of land use is no longer significant. Taken together, these analyses suggest that the two land uses have comparable FQI _adj_ at the average MAP mid-gradient, but may differ at the arid and mesic extremes. Since these conclusions were primarily driven by the outliers in the post-agricultural site, the results are suspect to caution (**Supplementary Table 6**).

### Community Analyses

**Figure 4:**
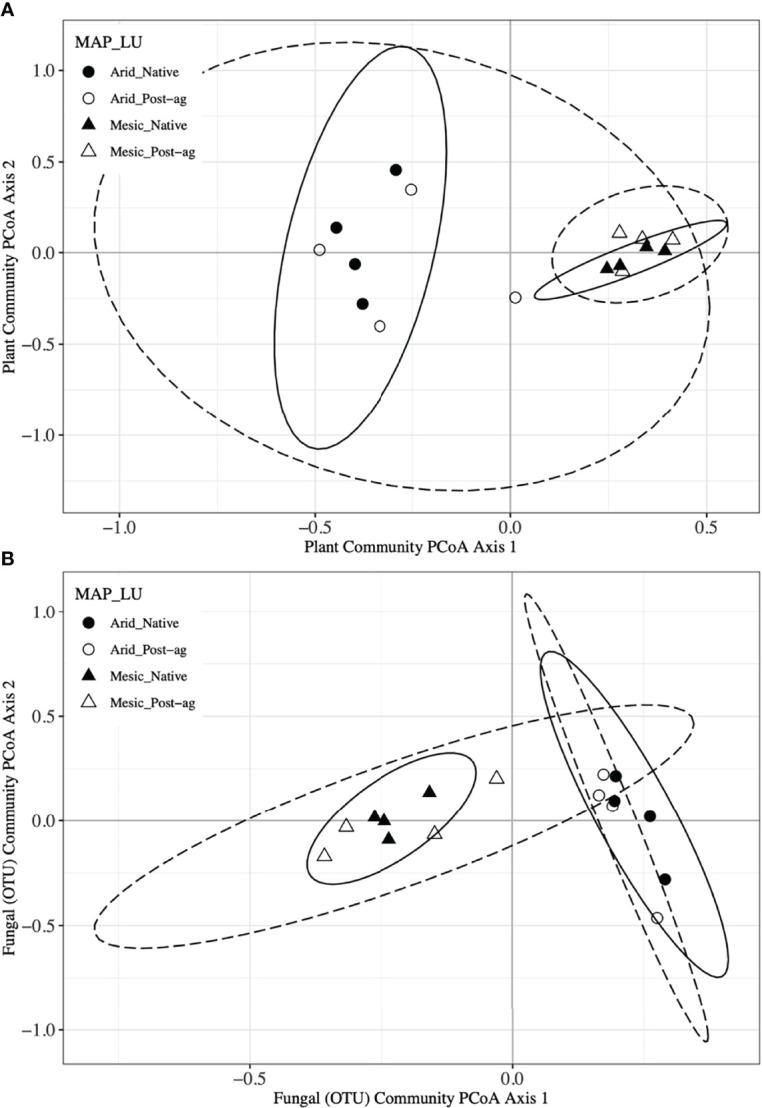
Principal Coordinates Analyses (PCoA) of plant **(A)** and fungal community composition using Operational Taxonomic Units (OTUs) **(B)** in native prairie remnants (solid line and filled symbols) and post-agricultural sites (dashed line and open symbols). Circles indicate the arid end of the precipitation gradient (455.7 – 634.9 mm yr^-1^), whereas triangles indicate the mesic end (760.9 – 1040.5 mm yr^-1^). Lines indicate the 95% confidence intervals around PCoA centroid for each group in the PCoA ordination.

We used PCoA and PERMANOVA to visualize and test for any community responses to MAP and land-use (**Figure 4**). In these analyses where we divided the MAP gradient into arid and mesic categories, we observed no evidence for interaction between MAP and land-use in either plant or fungal community composition (PERMANOVA: Plant: F_1,15_ = 0.99, R^2^ = 0.096, P = 0.375; OTU: F_1,15_ = 0.97, R^2^ = 0.058, P=0.506). However, both plant and fungal communities differed compositionally between the arid and mesic habitats (PERMANOVA: Plant: F_1,15_ = 5.77, R^2^ = 0.290, P = 0.001; OTU: F_1,15_ = 2.78, R^2^ = 0.166, P = 0.001). In contrast to many richness and diversity analyses, there was no evidence for difference in community composition between native prairie remnants and post-agricultural sites (PERMANOVA: Plant: F_1,15_ = 1.18, R^2^ = 0.059, P = 0.243; OTU: F_1,15_ = 1.02, R^2^ = 0.061, P = 0.395). In addition to our PERMANOVA analyses, in which we simply divided the precipitation gradient to arid and mesic habitats, we analyzed the PCoA axis scores using multiple linear regressions similar to those we used for community richness and diversity estimators. These models successfully predicted changes in composition and explained a substantial proportion of the variation in the first but not the second PCoA axis of both the plant and fungal communities (**Tables 2** and **3**; **Figures 2D, E** and **3D, E**). The first plant PCoA axis scores linearly increased, whereas the first fungal PCoA axis scores linearly decreased with MAP with no evidence for either land-use effects or interaction between the MAP and land-use (**Tables 2**, **3**; **Figures 2D** and **3D**). In contrast to the first PCoA axis, there was no evidence for MAP, land-use, or interaction effects for the second PCoA axis (**Tables 2**, **3**; **Figures 2E** and **3E**). This did not change when the potential low outlier (TRB_N) was removed (**Supplementary Table 6**).

To further explore differences in community composition and its responses to environmental and anthropogenic factors, we used constrained ordinations, distance-based redundancy analyses, using main effects of MAP, MAT, longitude, and LU for plant communities. We used similar analyses for fungal communities with the addition of the first plant PCoA axis to explain variation in fungal communities. These analyses further confirmed that climate variables (MAP and MAT) had a greater influence on plant and fungal community compositions than land-use. However, the environmental variables may be correlated as indicated by the similar direction of environmental vectors in ordination space (**Supplemental Figure 2**). To also assess the heterogeneity in plant and fungal community composition, we tested for community dispersion. Neither plant nor fungal communities differed in their dispersion between the arid and mesic habitats (Plant: F_1,15_ = 0.093, P = 0.783; OTU: F_1,15_ = 1.577, P = 0.199) or between native prairie remnants and post-agricultural sites (Plant: F_1,15_ = 1.18, P = 0.302; OTU: F_1,15_ = 3.264, P = 0.079).

To identify plant taxa that may underlie the observed community differences, we used indicator taxon analyses (De Caceres and Legendre, 2009) including fifty most abundant plant species. Our correction for false detection (FDR) proved conservative and resulted in the loss of all or most significant indicators. Consequently, we present both corrected and uncorrected values (**Supplementary Table 8**) for readers’ information. We identified eight arid and six mesic plant indicators before the FDR correction which highlight the transition from mixed grass to tallgrass prairie with increasing precipitation (**Supplementary Table 8**); one arid (*Pascopyrum smithii*) and two mesic (*Andropogon gerardii* and *Panicum virgatum*) indicators remained after the FDR correction. Before the FDR correction, arid indicators included six members of the family Poaceae including common mixed grass prairie taxa such as *Bromus japonicus*, *Bouteloua dactyloides*, and *Sporobolus cryptandrus*, as well as two members of the family Asteraceae (*Ambrosia psilostachya* and *Conyza canadensis*). Most indicators for mesic sites represented the family Poaceae and included the four dominant tallgrass prairie species: *Andropogon gerardii*, *Sorghastrum nutans*, *Panicum virgatum*, and *Schizachyrium scoparium* as well as *Sporobolus compositus.* One indicator represented the family Cyperaceae with various species of the genus *Carex* (**Supplementary Table 8**).

Similar indicator taxon analyses of the 100 most abundant fungal OTUs identified 17 arid and 14 mesic indicator OTUs before FDR correction (**Supplementary Table 9**). Indicators represented Phylum Ascomycota (15 arid and 11 mesic) and Basidiomycota (2 arid and 3 mesic). Eight arid and seven mesic indicators remained after FDR correction (arid: *Blumeria* sp., *Phaeoseptoriella zeae*, *Neostagonospora* sp., *Dinemasporium bambusicola*, *Gibberella tricincta*, *Alternaria* sp., *Cyphellophora* sp., and *Darksidea* sp.; mesic: *Phyllosticta sorghina*, Capnodiales sp., Herpotrichiellaceae sp., *Dissoconium* sp., Eurotiomycetes sp., *Neocosmospora falciformis*, and another Eurotiomycetes sp.). Many of the most abundant indicators were plant pathogens or other plant-associated fungi (**Supplementary Table 9**). Among the most abundant fungal indicators for arid sites was *Blumeria* sp., a member of the order Erysiphales (powdery mildews) which are obligate plant pathogens (Takamatsu, 2013); *Phaeoseptoriella zeae*, a foliar pathogen of *Zea mays* (Crous et al., 2019; Tennakoon et al., 2020); and *Neostagonospora* sp., a member of a genus of common pathogens of *Carex* (Quaedvlieg et al., 2013). Among the most abundant fungal indicators for the mesic sites was *Phyllosticta sorghina*, a common cereal crop pathogen (Rodrigo et al., 2018); *Dissoconium* sp. anamorph (teleomorph *Mycosphaerella*; Crous et al., 2007), a representative of a genus with many foliar pathogens (Li et al., 2012); and, a member of the family Herpotrichiellaceae, with many documented decomposers of plants or fungi (Untereiner and Malloch, 1999). Among indicators that were significant prior to FDR correction were *Puccinia andropogonis*, a common rust pathogen of the dominant grasses in the Great Plains (Szabo, 2006) and *Phyllozyma linderae* (basionym *Sporobolomyces linderae* Nakase, M. Takash. & Hamam.), a basidiomycetous phyllosphere yeast in the Phylum Pucciniomycotina, whose ecology remains elusive (Wang et al., 2015).

### Linkages Between the Plant and Fungal Communities

**Figure 5:**
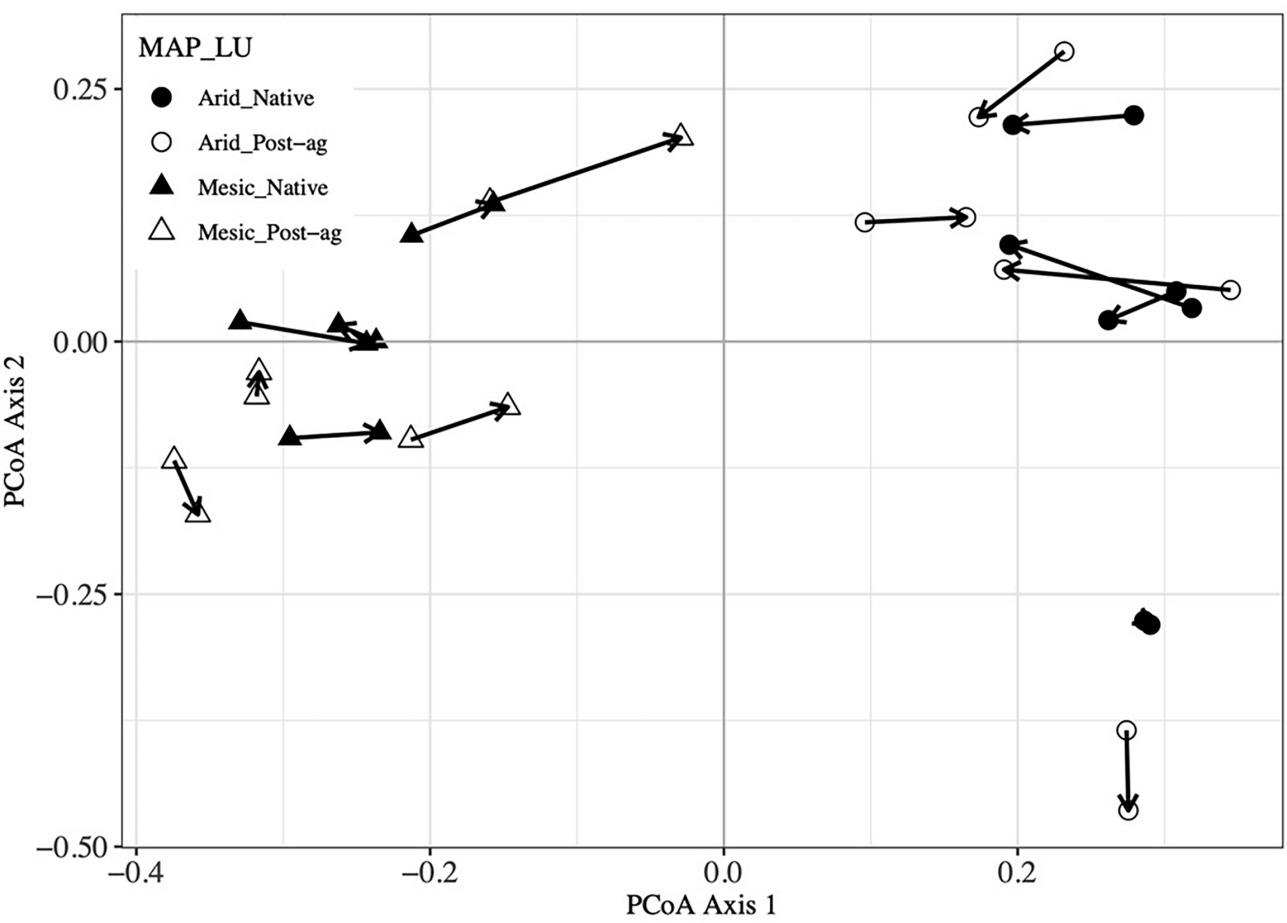
Procrustes analysis of plant community Principal Coordinates Analysis (PCoA) first and second ordination axes compared with Fungal Operational Taxonomic Unit (OTU) PCoA axes. Arrows point from plant community sample to the corresponding fungal community sample within a site.

Our co-located sampling of plant and fungal communities was designed to permit testing whether the two communities correlate. Our Mantel tests indicated that the Bray-Curtis distance matrices characterizing the community dissimilarities among the plots were highly correlated between the plant and fungal communities (R^2^ = 0.673, P = 0.001). Further, Mantel tests indicated that geographic distance did not correlate with plant communities (R^2^ = 0.078, P = 0.170) but correlated with fungal communities (R^2^ = 0.228, P = 0.024). Additionally, we utilized Procrustes analyses that compare two or more multidimensional shapes by translation, rotation and scaling the ordinations to maximize their superimposition (**Figure 5**). Corroborating the Mantel tests, these analyses highlighted the strong correlation between the plant and fungal two-dimensional PCoA ordinations (R^2^ = 0.573, P = 0.001).

## Discussion

We sampled the steep precipitation gradient in the central United States to better understand how plant and fungal communities vary with MAP, among native prairie remnants and post-agricultural sites, and how these communities may be linked. Our data indicate that both plant and fungal communities shift compositionally and increase in their diversity with MAP and had greater diversity in native remnant prairies than in post-agricultural sites. Further, although plant community richness also increased with MAP, fungal community richness did not. This lack of fungal richness response to MAP is surprising, given that the plant and fungal communities were correlated in composition. Although it is impossible to decouple MAP and other potential correlates, our analyses suggest the importance of MAP gradient and land-use history in controlling plant and fungal communities.

Our data supported our hypotheses that plant communities change in composition and increase in richness and diversity with MAP. Temperate grasslands in central North America range from 200 to 1200 mm·y^–1^ in MAP (Lauenroth et al., 1999) resulting in distinct ecosystems ranging from the shortgrass steppes with very low annual net primary productivity to the highly productive tallgrass prairies (Sala et al., 1988; Lauenroth et al., 1999). Our study covered a substantial proportion of this gradient (455.7–1040.5 mm yr^-1^) and our results are consistent with the transition from shortgrass steppes and mixed grass prairies to tallgrass prairies along the west-east precipitation gradient. The broad variability in MAP not only affects ecosystem annual net primary productivity, but also plant community composition, cover, and diversity (Lauenroth et al., 1978; Watson et al., 2021). Our results are congruent with Watson et al. (2021) and suggest that MAP is an important plant diversity predictor for regionally distinct plant communities. Our indicator taxon analyses highlighted that it is indeed the dominant graminoids that define these grassland communities, particularly so in the mesic tallgrass prairies.

Interestingly, our data suggested that floristic quality response to MAP depended on the land-use such that FQI_adj_ increased with MAP in native remnants but not in the post-agricultural fields. When we excluded the potential outliers, these responses became even more obvious and indicated an actual decline in FQIadj with MAP in the post-agricultural fields. The stochastic niche hypothesis (Tilman, 2004) predicts that plant communities with greater species richness would be less subject to establishment of new species – in our case also non-native species – than communities that have low species richness. This resistance to invasion is posited to stem more from resource exhaustion by the large number of potentially competing species with differing niches than from community diversity itself (McKane et al., 2002; Reich et al., 2012; Lannes et al., 2020). Plant species richness increased with MAP in both native prairie and post-agricultural sites in our analyses. As a result, our FQIadj results in the native prairie remnants seem consistent with this hypothesis but not in the post-agricultural sites. In contrast, in the post-agricultural sites, the decline in the FQI_adj_ in sites with greater species richness suggests that the agricultural land use legacy results in communities that are increasingly of lesser floristic quality and include a greater proportion of non-native species the greater the richness of comparable native sites is. It remains an open question whether the post-agricultural sites differ from the native prairies as a result of differences in available soil resources that reflect the past anthropogenic inputs during row crop production.

Plant communities and their shifts along gradients have been extensively studied (see Watson et al., 2021), whereas similar studies on fungal communities and/or their diversity are less common (but see *e.g.*, Tedersoo et al., 2014; Glynou et al., 2016; Rudgers et al., 2021). Factors that may affect fungal communities include latitude (Arnold et al., 2000; Arnold and Lutzoni, 2007; Tedersoo et al., 2014), climate (McGuire et al., 2012; U’ren et al., 2012; Zimmerman and Vitousek, 2012; Eusemann et al., 2016; Oita et al., 2021; Rudgers et al., 2021), soil (Tedersoo et al., 2020; Bowman and Arnold, 2021; Rudgers et al., 2021), plant host (Hoffman and Arnold, 2008; U’ren et al., 2012; Lau et al., 2013; Kembel and Mueller, 2014; Tedersoo et al., 2020; Rudgers et al., 2021), and disturbance (Delgado-Baquirizo et al., 2021). Some studies highlight strong host species and/or climatic/edaphic effects (*e.g*., Hoffman and Arnold, 2008; Tedersoo et al., 2020; Rudgers et al., 2021), whereas others find no support for correlations between plant community diversity and fungal communities (*e.g.*, McGuire et al., 2012; Tedersoo et al., 2014). While soil- and root-inhabiting fungal communities may be buffered against climatic drivers (Rudgers et al., 2021) or correlate with plant diversity (Shen et al., 2021), phyllosphere communities may be particularly sensitive to climatic drivers whilst buffered against edaphic factors (Bowman and Arnold, 2021; Oita et al., 2021). Consistent with our hypotheses and predictions, our data strongly suggest that phyllosphere fungal communities respond to MAP. These conclusions agree with others who have concluded that climatic factors strongly influence the phyllosphere fungal communities and their assembly (Carroll and Carroll, 1978; Zimmerman and Vitousek, 2012; U’Ren et al., 2012; Oita et al., 2021).

In addition to environmental factors, fungal communities respond to host species (Rudgers et al., 2021), although not necessarily to plant diversity or richness (McGuire et al., 2012; Tedersoo et al., 2014, but see Hooper et al., 2000; Shen et al., 2021). Our data clearly indicate that plant and fungal communities correlate, even though fungal richness neither strongly correlated with MAP nor was well predicted by climatic or plant community variables. Differences in plant metabolities and plant physiology may control phyllosphere community diversity and composition (Bailey et al., 2005; Rajala et al., 2014; Eusemann et al., 2016), resulting in greater fungal diversity in systems with greater plant diversity. We hypothesize that our observed compositional correlations likely stem from the niche heterogeneity provided by diverse plant communities that then may host diverse and distinct phyllosphere fungi. Indeed, some of our most common fungal indicator taxa were directly linked to their hosts, exemplified by foliar plant pathogens (*e.g*., *Phyllosticta sorghina* and *Blumeria* sp.). In sum, as host species communities shift, so does the probability of distinct fungal associates in the phyllosphere.

Ranking factors for their importance in structuring fungal communities is not simple. Some studies have suggested that edaphic factors can override the influence of host plant identity (Glynou et al., 2016), whereas others have suggested that the importance of edaphic factors varies among host species (Rudgers et al., 2021). In our study, MAP and plant community composition or diversity are inherently collinear and evaluating their relative importance in phyllosphere community assembly is therefore challenging. The controls may also differ among fungal guilds. McGuire et al. (2012) targeted lowland tropical rain forests with high plant richness in Panama and concluded that the compositionally distinct communities in soil and leaf litter differed in their compositional controls. (2012) targeted lowland tropical rain forests with high plant richness in Panama and concluded that the compositionally distinct fungal communities in soil and leaf litter differed in their compositional controls. Although the former correlated with MAP but not with plant richness, the latter correlated with neither MAP nor plant diversity. Further experiments that manipulated litter richness suggested that plant diversity may be less important in determining fungal richness than MAP as the fungal richness did not track the plant richness. In contrast to those studies, Shen et al. (2021) manipulated herbaceous plant community richness in a greenhouse experiment and concluded that the soil fungal richness correlated with that of the plant communities. Clearly, experimental systems, targeted fungal guilds and included host taxa appear essential controls of fungal communities. To better understand the relative importance of environmental factors and plant community estimators in the current experiment, we compared models using the main and interactive effects of land use and either MAP or plant estimators (richness, diversity, evenness, FQI_adj_, or PCoA axis 1). These simple model comparisons suggested that MAP is usually a superior predictor for fungal diversity and evenness. Although our studies emphasize the importance of climatic factors (see also Oita et al., 2021), further and more detailed studies may be needed to better resolve these issues. Understanding how climatic or edaphic variables can influence host-associated fungal communities is becoming increasingly important as the ongoing environmental change has the potential to disrupt host-microbe interactions (Ranelli et al., 2015; Glynou et al., 2016; Vetrovsky et al., 2019; Steidinger et al., 2020). Analysis of environmental gradients, such as MAP here, is a powerful approach to dissect such patterns (Rudgers et al., 2021).

Contrary to our hypotheses and predictions, we observed no strong evidence for differences in community composition and dispersion of plants or their phyllosphere fungi among the post-agricultural fields and native prairie remnants. However, our data indicate that native remnant prairies harbor greater plant richness and diversity as well as greater phyllosphere fungal diversity and evenness. Land-use and particularly its intensification have been posited as major drivers of biodiversity loss (Sala et al., 2000; Foley et al., 2005; Gossner et al., 2016; Brinkmann et al., 2019) and biotic and ecological homogenization (Gossner et al., 2016; Brinkmann et al., 2019; Delgado-Baquirizo et al., 2021). Some have suggested that the communities in post-agricultural sites remain distinct from those in native sites because of fungal dispersal limitations from native remnants (Turley et al., 2020), as has been reported for plants (Turley et al., 2017). The establishment of fungal communities in post-agricultural sites may also be a result of poor recovery of soil conditions after intensive agriculture (Bellemare et al., 2002; Dupouey et al., 2002; Flinn and Marks, 2007). Although the phyllosphere fungal communities correlate with phyllosphere chemistry and have been reported to differ among land-use types (e.g., Jumpponen and Jones, 2010), they may be less affected directly by the altered post-agricultural soil conditions than the soil- or root-inhabiting fungal communities are. Dispersal limitations for the phyllosphere communities may also be less restrictive than they are for soil-dwelling fungi (Bowman and Arnold, 2021). Our results are congruent with those of many others that emphasize agricultural legacy effects on bacterial and fungal communities decades after agricultural abandonment (Lauber et al., 2008; Upchurch et al., 2008; Jangild et al., 2011; Hui et al., 2018; Turley et al., 2020) as well as those that report strong biotic and ecological homogenization by anthropogenic land-use (*e.g.*, McKinney and Lockwood, 1999; Groffmann et al., 2014; Gossner et al., 2016; Delgado-Baquirizo et al., 2021; Kotze et al., 2021). Our data indicate that land-use is an important driver of phyllosphere communities across broad environmental gradients such as the steep precipitation gradient sampled here. Taken together, our study suggests that the phyllosphere communities in these systems closely track plant communities whose diversity has been impacted by the land-use legacies.

We simultaneously analyzed plant communities and their phyllosphere fungal communities to assess responses to MAP and land-use history across a precipitation gradient extending much of the known range of the temperate grasslands in the central Great Plains. Our data indicate strong climatic controls of both the plant and phyllosphere fungal communities and the lesser impact of the historic land-uses on community composition. Interestingly, these data highlight the resilience of the species-rich tallgrass prairies and comparatively lesser floristic quality of post-agricultural sites in the more mesic regions of this MAP gradient. The phyllosphere fungal communities also responded strongly to MAP, whereas the historic land-use appeared to have minimal to no effects on the compositionof these communities. However, our data indicate greater plant richness and diversity as well as greater fungal diversity and evenness in native remnant prairies than in post-agricultural sites. Although our model comparisons highlighted that MAP was commonly a stronger predictor of phyllosphere fungal community metrics than plant richness or community composition, the fungal communities closely tracked plant community composition suggesting that plant communities likely serve as a key driver for foliar fungal communities.

## Data Availability Statement

The datasets presented in this study can be found in online repositories. The names of the repository/repositories and accession number(s) can be found below: Sequence Read Archive (https://www.ncbi.nlm.nih.gov/sra/ ) under BioProject PRJNA795108, BioSamples SAMN24688311- SAMN24688331.

## Author Contributions

HD and AJ sampled the fungal communities, prepared samples for sequencing, conducted bioinformatic analyses, analysed data, and prepared manuscript. AU collected plant data and aided in manuscript revision. AK aided in sample collection, preparation for fungal sequencing, and manuscript revisions. GH prepared plant community data for analysis, estimated alpha diversity metrics, aided in plant community analysis and manuscript preparation and revisions. ST managed data in support of the site selection, and manuscript revisions. TL, TP, SL, and MG aided in site selection, sample collection and manuscript revision. All authors contributed to the article and approved the submitted version.

## Funding

NSF-EPSCoR award # 1656006 “Microbiomes of Aquatic, Plant and Soil Systems across Kansas (MAPS)”.

## Conflict of Interest

The authors declare that the research was conducted in the absence of any commercial or financial relationships that could be construed as a potential conflict of interest.

## Publisher’s Note

All claims expressed in this article are solely those of the authors and do not necessarily represent those of their affiliated organizations, or those of the publisher, the editors and the reviewers. Any product that may be evaluated in this article, or claim that may be made by its manufacturer, is not guaranteed or endorsed by the publisher.
