# Supplementary material for: Precipitation, Not Land Use, Primarily Determines the Composition of Both Plant and Phyllosphere Fungal Communities
Source: Front Fungal Biol. 2022 Jul 7;3:805225. doi: 10.3389/ffunb.2022.805225 (PMC10512219; doi:10.3389/ffunb.2022.805225)
Supplement: Supplementary file 1 [file DataSheet_1.docx]

# **Results of ASV analyses**

## **Community descriptions**

Following quality control and removal of rare sequences, we retained a total of 3,246,656 high quality sequences that clustered into 9,760 ASVs. The number of retained sequences differed between ASV and OTU pipelines because of the larger number of rare ASVs that were removed. The sequencing yields ranged from 95,925 to 359,387 per sample with a mean yield of 202,916 ± 90,823.6 (SD). The ASVs, their observed frequencies, and taxonomic assignments are listed in Supplemental Tables 10 and 11.

Our ASV data were dominated by the Phylum Ascomycota (63.7% sequences and 59.0% ASVs), the Phylum Basidiomycota (16.0% sequences and 15.6 % ASVs), and a fairly large portion of unidentified taxa (18.1% sequences and 14.8 % ASVs), followed by the Phylum Glomeromycota (1.2% sequences and 6.9% ASVs), Chytridiomycota (0.4% sequences and 2.2% ASVs), and several Phyla that made up <1% of sequences and ASVs (Mortierellomycota, Mucoromycota, Kickxellomycota, Rozellomycota, Olpidiomycota, Entorrhizomycota, Aphelidiomycota, Entomophthoromycota, Aphelidiomycota, Entomophthoromycota, Calcarisporiellomycota, and Blastocladiomycota). Relative abundance of fungal Orders can be found in Supplemental Figure 1B. ASV were assigned to a total of 795 genera. a large proportion of ASVs were not assigned to the level of a genus – 3,783 ASVs (38.7%). Among those with genus level assignments, the most abundant were *Alternaria* with 37 ASVs (4.0% of all sequences and > 0.4% of all ASVs) followed by *Cladosporium* with 86 ASVs (3.5% sequences), and *Dissoconium* with 102 ASVs (2.9% sequences). The ten most abundant genera were common phyllosphere inhabitants including *Alternaria*, *Dissoconium*, *Phaeosphaerea*, *Puccinia*, *Fusarium*, *Blumeria*, and *Aureobasidium*.

## **Alpha diversity and regression analyses**

## Our regression model — using MAP normalized around the mean precipitation (730.01 mm yr^-1^), LU, and their interaction as predictors — poorly predicted fungal richness (S_Obs_) and explained only a small proportion of the variation. These analyses provided no evidence for fungal richness responses to MAP, LU, or their interaction (Supplemental Table 12; Supplemental Figure 3A). This result did not change whether or not the potential outlier (LVN_N) was excluded from the analysis (Supplemental Table 6). AIC comparisons suggest that plant predictors or geographic distance were not superior to MAP (Supplemental Table 7) except in the case of FQI_adj_ which was a better predictor for ASV richness (F_3,12_ = 1.28, R^2^_adj_ = 0.054, P = 0.324), however, none of these models performed well in predicting fungal richness overall.

Our regression models — using MAP normalized around the mean precipitation (730.01 mm yr^-1^), LU, and their interaction as predictors — predicted fungal diversity (H’) and explained a large proportion of the variation in their communities (Supplemental Table 12; Supplemental Figure 3B). There was evidence for interaction between MAP and LU where fungal diversity increased with MAP in the native prairie remnants but did not significantly change with increasing MAP in post-agricultural sites. There was also evidence for a land-use main effect that indicated greater fungal diversity in native prairie remnants than post-agricultural sites (Supplemental Table 12; Supplemental Figure 3B). AIC comparisons suggest that replacing MAP with geographic distance or plant community metrics did not result in a superior model for predicting fungal diversity (Supplemental Table 7).

Our regression models – using MAP normalized around the mean precipitation (730.01 mm yr^-1^), LU, and their interaction as predictors – predicted fungal community evenness (E_H_) and explained much of its variation (Supplemental Table 12; Supplemental Figure 3C). Fungal evenness increased with MAP in native prairies (significantly for ASVs, but only marginally significantly for OTUs), but did not change in post-agricultural sites. There was also evidence for a land-use main effect indicating greater fungal evenness in native remnant prairies than post-agricultural sites (Table 3; Figure 3C). (Supplemental Table 12; Supplemental Figure 3C). AIC comparisons suggest that replacing MAP with geographic distance or plant community metrics did not result in a superior model for predicting fungal evenness (Supplemental Table 7).

## **Community analyses**

We used PCoA and PERMANOVA to visualize and test for any community responses to MAP and land-use (Supplemental Figure 4). In these analyses, we observed no evidence for interaction between MAP and land-use in either plant or fungal community composition (PERMANOVA: F_1,15_ = 0.91, R^2^ = 0.0557, P = 0.647). However, both plant and fungal communities differed compositionally between the arid and mesic habitats (PERMANOVA: F_1,15_ = 2.44, R^2^ = 0.150, P = 0.001). Similar to the richness and diversity analyses, there was no evidence for difference in community composition between native prairies remnants and post-agricultural sites (PERMANOVA: Plant: F_1,15_ = 0.92, R^2^ = 0.056, P = 0.637). In addition to our PERMANOVA analyses, in which we simply divided the precipitation gradient to arid and mesic habitats, we analyzed the PCoA axis scores for the plant and ASV-inferred fungal communities using multiple linear regressions similar to those we used for community richness and diversity estimators. These models successfully predicted and explained a substantial proportion of the variation in the first but not the second PCoA axis of both the plant and fungal communities (Supplemental Table 12; Supplemental Figure 3D-E). PCoA axis 1 scores linearly decreased with MAP with no evidence for either land-use effects or interaction between the MAP and land-use (Supplemental Table 12; Supplemental Figure 3D). In contrast to PCoA axis 1, there was no evidence for MAP, land-use, or interaction for PCoA axis 2 (Supplemental Table 12; Supplemental Figure 3E). This did not change when the potential low outlier (TRB_N) was removed (Supplemental Table 6).

To further explore differences in community composition and its responses to environmental and anthropogenic factors, we used constrained ordinations, distance-based redundancy analyses, using main effects of MAP normalized around the mean precipitation (730.01 mm yr^-1^), MAT, longitude, LU, and first plant PCoA axis to explain variation in fungal communities. These analyses further confirmed that climate variables (MAP and MAT) had a greater influence on fungal community compositions than land-use. However, these environmental variables may be correlated as indicated by the similar direction of environmental vectors arrows in ordination space (Supplemental Figure 5).

To also assess the heterogeneity in plant and fungal community composition, we tested community dispersion. Neither plant nor fungal communities differed in their dispersion between the arid and mesic habitats (F_1,15_ = 1.585, P = 0.216) or between native prairie remnants and post-agricultural sites (F_1,15_ = 0.843, P = 0.349).

Indicator taxon analyses of the 200 most abundant fungal ASVs identified 33 arid and 28 mesic indicator ASVs before FDR correction (Supplemental Table 13). Indicators represented Phylum Ascomycota (30 arid and 22 mesic ASVs) and Basidiomycota (3 arid and 5 mesic ASVs). One mesic indicator represented unclassified fungi or could not be assigned beyond Kingdom Fungi. Fifteen arid and eight mesic indicators remained after FDR correction (arid: *Paraphaeosphaeria* sp., *Phaeosphaeria* sp., *Phaeosphaeriaceae* sp., *Saitozyma paraflava*, *Phaeopoacea* sp., *Cyphellophora* sp, *Blumeria* sp., Ascomycota sp., *Phaeoseptoriella zeae*, *Didymellaceae* sp., *Neostagonospora* sp., another *Blumeria* sp., *Dinemasporium bambusicola*, *Ascochyta hordei*, and *Alternaria* sp.; mesic: *Epicoccum sorghinum*, Capnodiales sp., *Codinaea* sp., *Phaeopoacea* sp., *Neoascochyta* sp., *Phaeosphaeria* sp., *Dissoconium* sp., *Symmetrospora gracilis*). Many of the most abundant indicators were plant pathogens or other plant-associated fungi (Supplemental Table 13). Among the most abundant fungal indicators for arid sites were two *Blumeria* sp., a member of the order Erysiphales (powdery mildews) which are obligate plant pathogens (Takamatsu 2013); *Phaeoseptoriella zeae,* a foliar pathogen of *Zea mays* (Crous et al., 2019, Tennakoon et al., 2020); and *Neostagonospora* sp. common pathogens of Carex (Quaedvlieg et al., 2013). Among the most abundant fungal indicators for the mesic sites was *Epicoccum sorghinum*, a common cereal crop pathogen (Rodrigo et al., 2018) and *Dissoconium* sp. anamorph (teleomorph *Mycosphaerella*; Crous et al., 2007), a representative of a genus with many foliar pathogens (Li et al., 2012). Among those that were significant prior to FDR correction was a member of the family Herpotrichiellaceae, with many documented decomposers of plants or fungi (Untereiner and Malloch, 1999). Some mesic indicators that were significant in our OTU analyses before FDR correction such as *Puccinia andropogonis*, a common rust pathogen of the dominant grasses in the Great Plains (Szabo, 2006) and *Phyllozyma linderae* (basidionym *Sporobolus linderae*), a basidiomycetous phyllosphere yeast in the Phylum Pucciniomycotina, whose ecology remains elusive (Wang et al., 2015) were marginally significant in ASV analyses before FDR correction (Supplemental Table 13).

## **Linkages between the plant and fungal communities**

Our co-located sampling of plant and fungal communities was designed to permit testing whether the two communities correlate. Our Mantel tests indicated that the Bray-Curtis distance matrices characterizing the community dissimilarities among the plots were highly correlated between the plant and ASV-inferred fungal communities (R^2^ = 0. 644, P = 0.001). Additionally, we utilized Procrustes analyses that compare two or more multidimensional shapes by translation, rotation and scaling the ordinations to maximize their superimposition (Supplemental Figure 6). Corroborating the Mantel tests, these analyses highlighted the strong correlation between the plant and ASV-based fungal two-dimensional PCoA ordinations (R^2^ = 0. 768, P = 0.001).
