# Supplementary material for: Precipitation, Not Land Use, Primarily Determines the Composition of Both Plant and Phyllosphere Fungal Communities
Source: Front Fungal Biol. 2022 Jul 7;3:805225. doi: 10.3389/ffunb.2022.805225 (PMC10512219; doi:10.3389/ffunb.2022.805225)
Supplement: Supplementary file 2 [file DataSheet_2.docx]

Supplementary Material

# Supplementary Figures


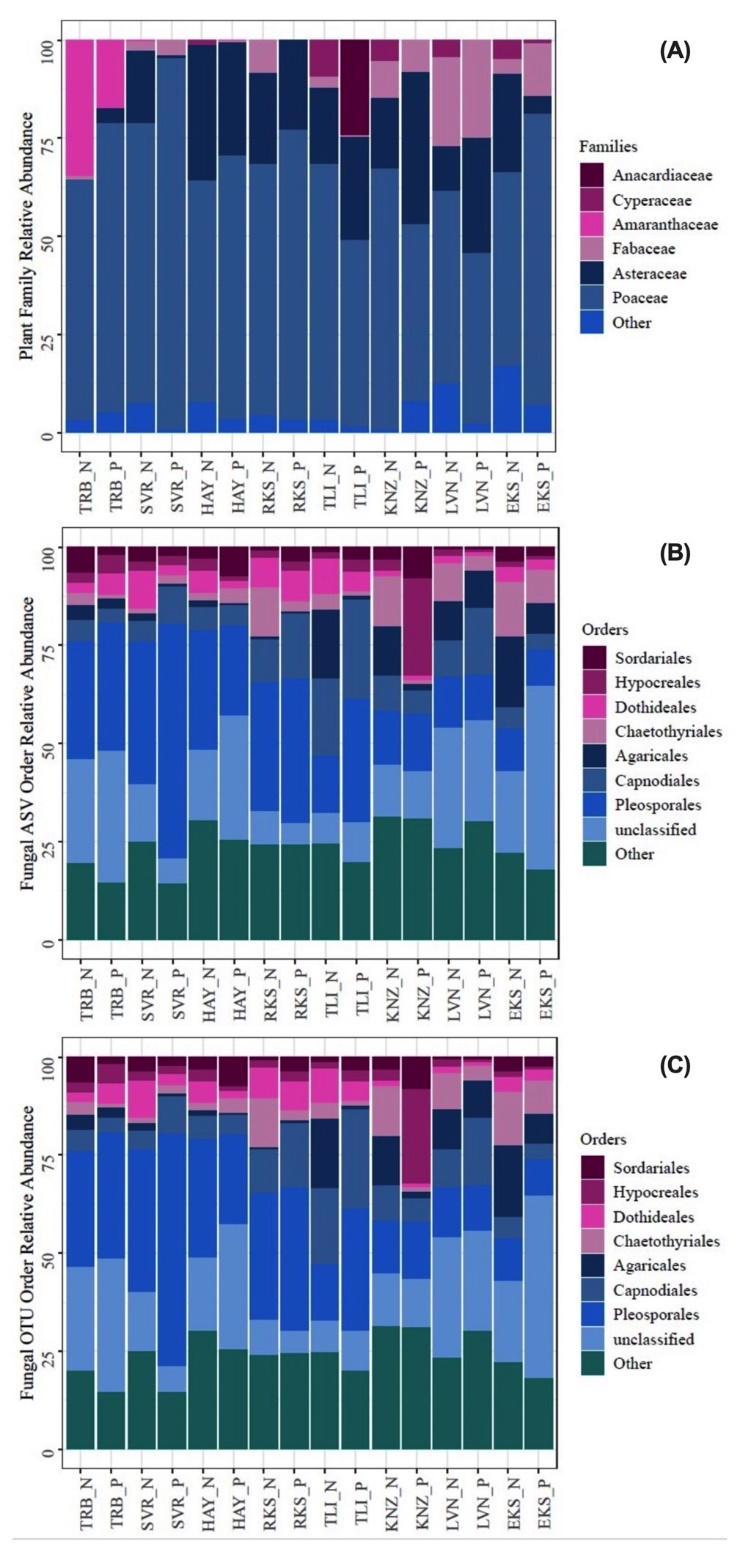


**Supplementary Figure 1.** Relative abundance of plant families **(A)**, fungal Amplicon Sequence Variants (ASVs) order assignment **(B)**, and Operational Taxonomic Units (OTUs) order assignment **(C)**. Plant families representing less than 1% and fungal orders representing less than 2% of the entire dataset are listed as “Other”.


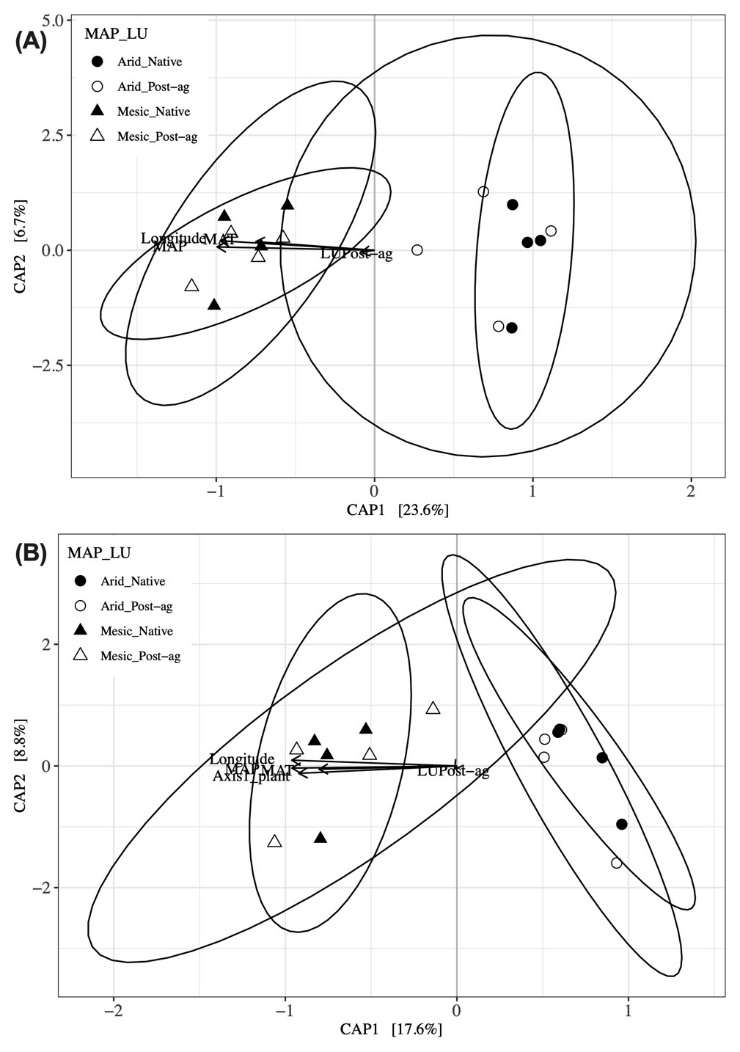


**Supplementary Figure 2.** Distance Based Redundancy Analysis of the Bray-Curtis Distance of plant community (F_4,11_ = 1.80, P = 0.006) **(A)** and phyllosphere fungal Operational Taxonomic Units (OTUs) (F_5,10_ = 1.60, P = 0.001) **(B)**. Main effects including Longitude (Plant: F_1,11_ = 1.21, P = 0.223; OTU: F_1,10_ = 1.58, P = 0.018), Mean Annual Precipitation (MAP) normalized around the mean precipitation (730.01 mm yr^-1^) (Plant: F_1,11_ = 2.24 , P = 0.026; OTU: F_1,10_ = 1.58, P = 0.018), Mean Annual Temperature (MAT) (Plant: F_1,11_ =2.76, P = 0.011; OTU: F_1,10_ = 2.43, P = 0.001) and land-use (LU) (Plant: F_1,11_ = 0.97, P = 0.389; OTU: F_1,10_ = 1.06, P = 0.339) were used in models for both plant and fungal communities. The first Plant PCoA axis (OTU: F_1,10_ = 1.35, P = 0.064) was additionally included for fungal communities. Arrows indicate the correlation of environmental variables with community composition.


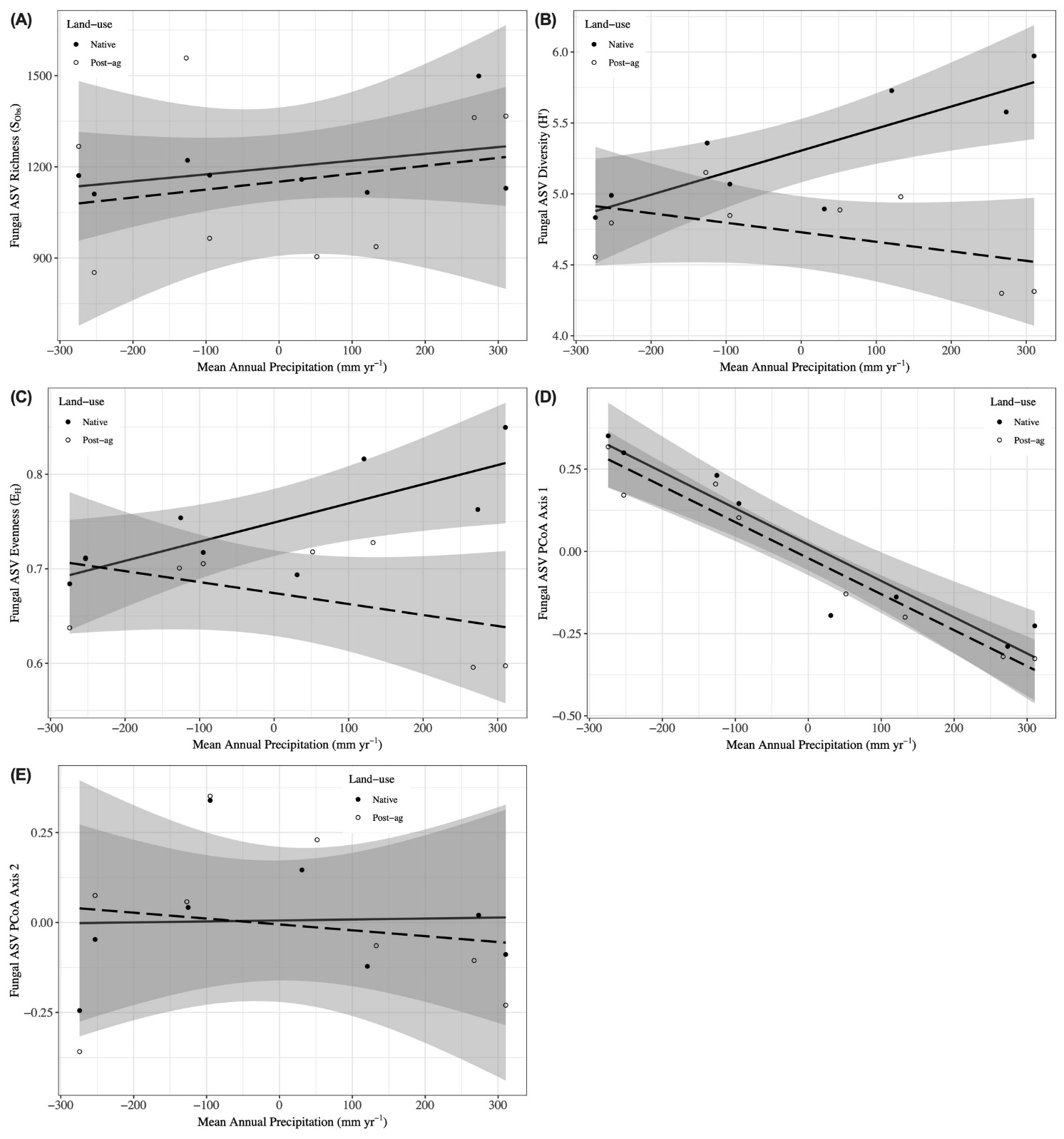


**Supplementary Figure 3.** Fungal Amplicon Sequence Variant (ASV) responses to mean annual precipitation (MAP) in native prairie remnants (solid line and filled symbols) and post-agricultural sites (dashed line and open symbols). Models predict observed species richness (S_Obs_) **(A)**, Shannon diversity (H’) **(B)**, evenness (E_H_) **(C)**, PCoA Axis 1 scores **(D)**, PCoA Axis 2 scores **(E)**. The shaded areas represent 95% confidence intervals around the model predictions.


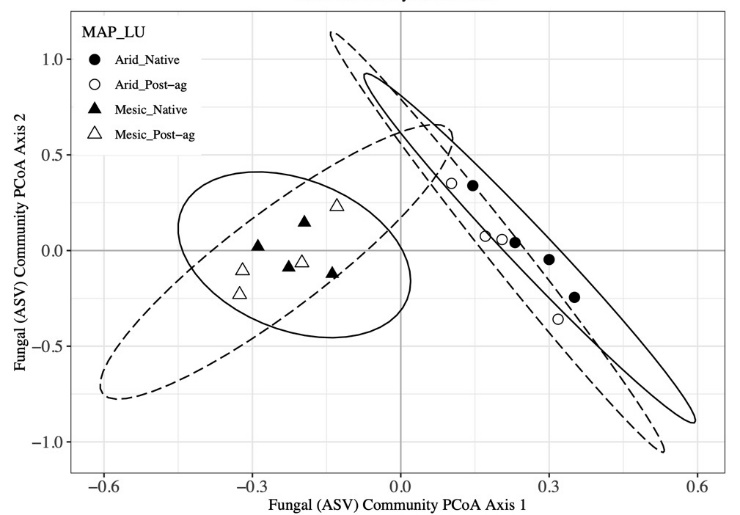


**Supplementary Figure 4.** Principal Coordinates Analyses (PCoA) of fungal community composition using Amplicon Sequence Variants (ASVs) in native prairie remnants (solid line and filled symbols) and post-agricultural sites (dashed line and open symbols). Circles indicate the arid end of the precipitation gradient (455.7 – 634.9 mm yr^-1^), whereas triangles indicate the mesic end (760.9 – 1040.5 mm yr^-1^). Lines indicate the 95% confidence intervals around PCoA centroid for each group in the PCoA ordination.


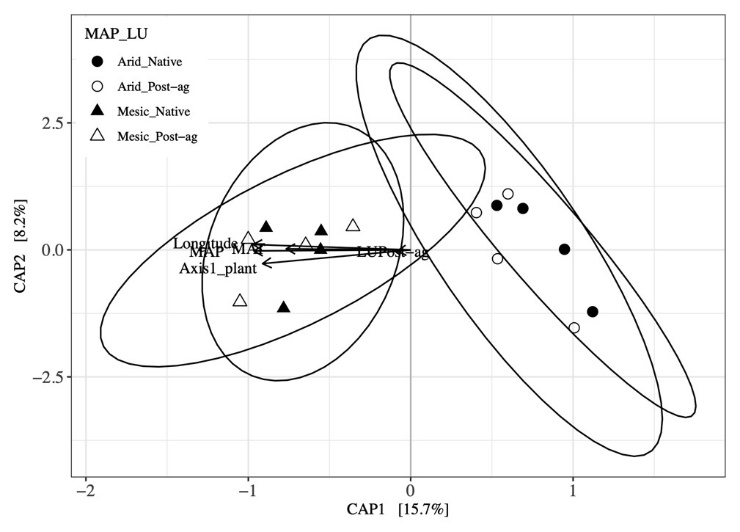


**Supplementary Figure 5.** Distance Based Redundancy Analysis of the Bray-Curtis Distance of fungal Amplicon Sequence Variants (ASVs; F_5,10_ = 1.42, P = 0.001). Main effects including Longitude (ASV: F_1,10_ = 1.38, P = 0.045), Mean Annual Precipitation (MAP) normalized around the mean precipitation (730.01 mm yr^-1^) (ASV: F_1,10_ = 1.54, P = 0.017), Mean Annual Temperature (MAT) (ASV: F_1,10_ = 1.98, P = 0.002) and land-use (LU) (ASV: F_1,10_ = 0.94, P = 0.595) and first plant PCoA axis (ASV: F_1,10_ = 1.25, P = 0.091) were used in the model. Arrows indicate the correlation of environmental variables with community composition.


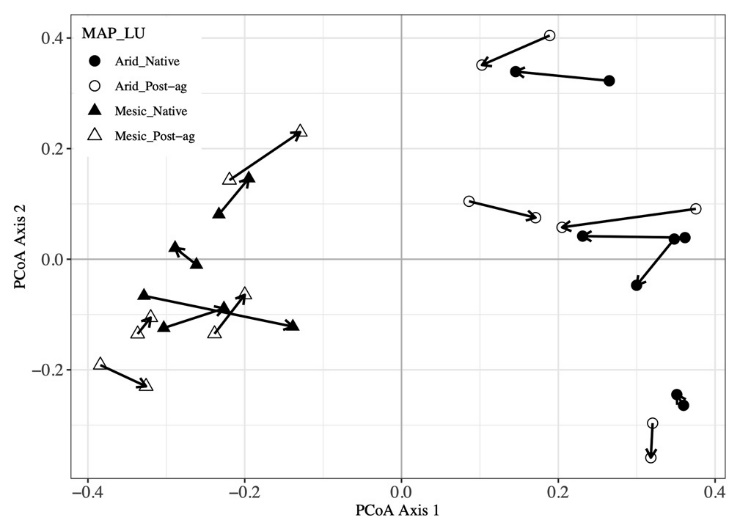


**Supplementary Figure 6.** Procrustes analysis of plant community Principal Coordinate Analysis (PCoA) first and second ordination axes compared with Fungal Amplicon Sequence Variant (ASV) PCoA axes. Arrows point from plant community sample to the corresponding fungal community sample within a site.
