# Supplementary material for: Precipitation, Not Land Use, Primarily Determines the Composition of Both Plant and Phyllosphere Fungal Communities
Source: Front Fungal Biol. 2022 Jul 7;3:805225. doi: 10.3389/ffunb.2022.805225 (PMC10512219; doi:10.3389/ffunb.2022.805225)
Supplement: Supplementary file 7 [file Table_5.docx]

**Supplementary Table 5.** Multiple linear regression model adjusted R-squared (R^2^_adj_) and Akaike's Information Criteria (AIC) for plant community estimators predicted by land use history (LU) and listed main effect predictors and their interaction (LU x predictor) with native prairie as reference (0) compared to post-agricultural site (1).

| Predictor | Plant Richness (S_obs_) | Plant Diversity (H') | Plant Evenness (E_H_) | Plant FQI _adj_ |
| --- | --- | --- | --- | --- |
| Longitude (DD) | R^2^_adj_ = 0.67; AIC= 107.10 | R^2^_adj_ = 0.52; AIC= 23.16 | R^2^_adj_ =–0.06; AIC=–36.74 | R^2^_adj_ = 0.39; AIC= 124.03 |
| MAP (mm yr^-1^) | R^2^_adj_ = 0.64; AIC= 107.85 | R^2^_adj_ = 0.51; AIC= 23.61 | R^2^_adj_ =–0.10; AIC=–36.28 | R^2^_adj_ = 0.41; AIC= 123.44 |
