# Supplementary material for: Precipitation, Not Land Use, Primarily Determines the Composition of Both Plant and Phyllosphere Fungal Communities
Source: Front Fungal Biol. 2022 Jul 7;3:805225. doi: 10.3389/ffunb.2022.805225 (PMC10512219; doi:10.3389/ffunb.2022.805225)
Supplement: Supplementary file 8 [file Table_6.docx]

**Supplementary Table 6.** Multiple linear regression model statistics for plant and fungal community estimators predicted by land use history (LU) and mean annual precipitation (MAP) normalized around the mean precipitation (730.01 mm yr^-1^) main effects and their interaction (LU x MAP) with native prairie as reference (0) compared to post-agricultural site (1). Statistically significant models and predictors (P<0.05) are bold-faced. Parameter estimate significances are denoted as 'ns' for not significant, ‘(*)’ for 0.05≤P<0.10, ‘*’ for 0.01≤P<0.05, ‘**’ for 0.001≤P<0.01, and ‘***’ for P<0.001. Response variables with outliers were analyzed with and without the identified outliers. Models shown here do not include potential outliers.

| Response | Model | Predictor | Estimate±SE | \|t –value\| |
| --- | --- | --- | --- | --- |
| Plant FQI_adj_ | **F_3,11_ =9.57^*^, R^2^_adj_ =0.647, AIC=103.47** | **Intercept** | **31.78±2.25** | **14.11^***^** |
|  |  | Land Use (LU) | –5.15±3.34 | –1.54^ns^ |
|  |  | **MAP** | **5.16x10^-2^±1.08x10^-2^** | **4.79^***^** |
|  |  | **LU x MAP** | **–7.13x10^-2^±1.64x10^-2^** | **–4.35^**^** |
|  |  |  |  |  |
| Plant Diversity (H') | **F_3,11_ =8.00^*^, R^2^_adj_=0.600, AIC=18.15** | **Intercept** | **2.54±1.43x10^-1^** | **17.78^***^** |
|  |  | **LU** | **–6.80x10^-1^ ±1.94x10^-1^** | **–3.51^**^** |
|  |  | MAP | 9.34x10^-4^±7.20x10^-4^ | 1.30^ns^ |
|  |  | LU x MAP | 8.91x10^-4^±9.53x10^-4^ | 0.94^ns^ |
|  |  |  |  |  |
| Plant PCoA Axis 2 | F_3,12_ =0.11^ns^, R^2^_adj_=–0.237, AIC= 0.39 | Intercept | –4.94x10^-2^±7.89x10^-2^ | –0.63^ns^ |
|  |  | LU | 3.27x10^-2^±1.07x10^-1^ | 0.31^ns^ |
|  |  | MAP | 8.24x10^-5^±3.98x10^-4^ | 0.21^ns^ |
|  |  | LU x MAP | 6.93x10^-5^±5.27x10^-4^ | 0.13^ns^ |
|  |  |  |  |  |
| ASV Richness (S_Obs_) | F_3,11_=0.19^ns^, R^2^_adj_= –0.21, AIC=208.31 | **Intercept** | **1151.76±81.06** | **14.21^***^** |
|  |  | LU | –5.07x10^-1^±109.90 | –0.01^ns^ |
|  |  | MAP | –6.02x10^-2^±4.08x10^-1^ | –0.15^ns^ |
|  |  | LU x MAP | 3.21x10^-1^±5.40x10^-1^ | 0.59^ns^ |
|  |  |  |  |  |
| OTU Richness (S_Obs_) | F_3,11_=0.73^ns^, R^2^_ad_**_j_**= –0.061, AIC=189.33 | **Intercept** | **755.85±43.05** | **17.56^***^** |
|  |  | LU | –59.19±58.37 | –1.01^ns^ |
|  |  | MAP | –2.38x10^-2^±2.17x10^-1^ | –0.11^ns^ |
|  |  | LU x MAP | 2.21x10^-1^±2.87x10^-1^ | 0.77^ns^ |
