# Supplementary material for: Precipitation, Not Land Use, Primarily Determines the Composition of Both Plant and Phyllosphere Fungal Communities
Source: Front Fungal Biol. 2022 Jul 7;3:805225. doi: 10.3389/ffunb.2022.805225 (PMC10512219; doi:10.3389/ffunb.2022.805225)
Supplement: Supplementary file 9 [file Table_7.docx]

**Supplementary Table 7.** Multiple linear regression model adjusted R-squared (R^2^_adj_) and Akaike's Information Criteria (AIC) for fungal community estimators predicted by land use history (LU) and listed predictor main effects and their interaction (LU x predictor) with native prairie as reference (0) compared to post-agricultural site (1). Superior models (ΔAIC > 2; Burnham and Anderson, 2004) are bold faced. If no model was superior, we used the model LU x MAP.

| Predictor | ASV richness (S_obs_) | OTU Richness (S_obs_) | ASV Diversity (H') | OTU Diversity (H') | ASV Evenness (E_H_) | OTU Evenness (E_H_) |
| --- | --- | --- | --- | --- | --- | --- |
| Plant Richness (S_obs_) | R^2^_adj_ =–0.05; AIC=221.76 | **R^2^_adj_ = 0.22; AIC=199.43** | R^2^_adj_ =0.47; AIC=16.20 | R^2^_adj_ =0.39; AIC=17.74 | R^2^_adj_ =0.32; AIC=–41.80 | R^2^_adj_ =0.24; AIC=–40.27 |
| Plant Diversity (H') | R^2^_adj_ =–0.02; AIC=221.18 | R^2^_adj_ = 0.11; AIC=201.64 | R^2^_adj_ =0.43; AIC=17.16 | R^2^_adj_ =0.38; AIC=18.07 | R^2^_adj_ =0.31; AIC=–41.59 | R^2^_adj_ =0.25; AIC=–40.48 |
| Plant Evenness (E_H_) | R^2^_adj_ =–0.19; AIC=223.73 | R^2^_adj_ = 0.01; AIC=203.29 | R^2^_adj_ =0.28; AIC=20.95 | R^2^_adj_ =0.30; AIC=19.93 | R^2^_adj_ =0.20; AIC=–39.11 | R^2^_adj_ =0.18; AIC=–39.07 |
| Plant PCoA Axis 1 | R^2^_adj_ =–0.18; AIC=223.60 | R^2^_adj_ =–0.05; AIC=204.30 | R^2^_adj_ =0.51; AIC=14.83 | R^2^_adj_ =0.46; AIC=15.78 | R^2^_adj_ =0.36; AIC=–42.72 | R^2^_adj_ =0.33; AIC=–42.14 |
| Plant FQI_adj_ | **R^2^_adj_ = 0.05; AIC=220.03** | R^2^_adj_ =–0.04; AIC=204.14 | R^2^_adj_ =0.52; AIC=14.69 | R^2^_adj_ =0.53; AIC=13.69 | R^2^_adj_ =0.45; AIC=–45.16 | R^2^_adj_ =0.45; AIC=–45.25 |
| Longitude (DD) | R^2^_adj_ =–0.15; AIC=223.18 | R^2^_adj_ =0.05 ; AIC=202.59 | R^2^_adj_ =0.61; AIC=11.38 | R^2^_adj_ =0.50; AIC=14.41 | R^2^_adj_ =0.46; AIC=–45.53 | R^2^_adj_ =0.38; AIC=–43.55 |
| MAP (mm yr^-1^) | R^2^_adj_ =–0.15; AIC=223.15 | R^2^_adj_ = 0.03; AIC=202.93 | **R^2^_adj_ =0.65; AIC= 9.63** | R^2^_adj_ =0.54; AIC=13.21 | **R^2^_adj_ =0.51; AIC=–47.00** | R^2^_adj_ =0.43; AIC=–44.69 |
