# Supplementary material for: Precipitation, Not Land Use, Primarily Determines the Composition of Both Plant and Phyllosphere Fungal Communities
Source: Front Fungal Biol. 2022 Jul 7;3:805225. doi: 10.3389/ffunb.2022.805225 (PMC10512219; doi:10.3389/ffunb.2022.805225)
Supplement: Supplementary file 14 [file Table_12.docx]

**Supplementary Table 12.** Multiple linear regression model statistics for fungal Amplicon Sequence Variant (ASV) community diversity, richness evenness and compositional estimates predicted by land use history (LU) and mean annual precipitation (MAP) main effects and their interaction (LU x MAP) with native prairie as reference (0) compared to post-agricultural prairie (1). Statistically significant models and predictors (P<0.05) are bold-faced. Parameter estimate significances are denoted as 'ns' for not significant, ‘(*)’ for 0.05≤P<0.10, ‘*’ for 0.01≤P<0.05, ‘**’ for 0.001≤P<0.01, and ‘***’ for P<0.001. Response variables with outliers were analyzed with and without the identified outliers. Models shown here include potential outliers, Supplemental Table 5 provides model details with outliers removed.

| Response | Model | Predictor | Estimate±SE | \|t-value\| |
| --- | --- | --- | --- | --- |
| ASV Richness (S_Obs_)^[[1]](#footnote-1)^ | F_3,12_ =0.35^ns^, R^2^_adj_=–0.15, AIC=223.15 | **Intercept** | **1197.63±77.17** | **15.52^***^** |
|  |  | LU | –46.38±109.13 | –0.43^ns^ |
|  |  | MAP | 2.24x10^-1^±3.69x10^-1^ | 0.61^ns^ |
|  |  | LU x MAP | 3.61x10^-2^±5.21x10^-1^ | 0.07^ns^ |
|  |  |  |  |  |
| ASV Diversity (H') | **F_3,12_ =10.15^*^, R^2^_adj_=0.647, AIC=9.63** | **Intercept** | **5.31±9.77x10^-2^** | **54.32^***^** |
|  |  | **LU** | **–5.76x10^-1^±1.38x10^-1^** | **–4.17^**^** |
|  |  | **MAP** | **1.55x10^-3^±4.67x10^-4^** | **3.33^**^** |
|  |  | **LU x MAP** | **–2.23x10^-3^±6.59x10^-4^** | **–3.38^**^** |
|  |  |  |  |  |
| ASV Evenness (E_H_) | **F_3,12_ =6.23^*^, R^2^_adj_=0.511, AIC= –47.00** | **Intercept** | **7.49x10^-1^±1.66x10^-2^** | **45.02^***^** |
|  |  | **LU** | **–7.46x10^-2^±2.35x10^-2^** | **–3.17^**^** |
|  |  | **MAP** | **2.03x10^-4^±7.95x10^-5^** | **2.55^*^** |
|  |  | **LU x MAP** | **–3.19x10^-4^±1.12x10^-4^** | **–2.84^*^** |
|  |  |  |  |  |
| ASV PCoA Axis 1 | **F_3,12_ =48.40^***^, R^2^_adj_=0.905, AIC= –31.32** | Intercept | 2.06x10^-2^±2.72x10^-2^ | 0.76^ns^ |
|  |  | LU | –4.12x10^-2^±3.84x10^-2^ | –1.07^ns^ |
|  |  | **MAP** | **–1.10x10^-3^±1.30x10^-4^** | **–8.50^***^** |
|  |  | LU x MAP | –8.42x10^-6^±1.83x10^-4^ | 0.05^ns^ |
|  |  |  |  |  |
| ASV PCoA Axis 2 | F_3,12_ =0.07^ns^, R^2^_adj_=–0.229, AIC= 2.75 | Intercept | 5.66x10^-3^±7.88x10^-2^ | 0.07^ns^ |
|  |  | LU | –1.10x10^-2^±1.11x10^-1^ | –0.10^ns^ |
|  |  | MAP | 2.68x10^-5^±3.76x10^-4^ | 0.07^ns^ |
|  |  | LU x MAP | –1.90x10^-4^±5.31x10^-4^ | –0.36^ns^ |
|  |  |  |  |  |

1. Contained a high outlier in LVN_N retained in this analysis [↑](#footnote-ref-1)
